# Sarbecoviruses Suppress Innate Immunity by Recruiting PPM1A to Modulate STAT2 and ORF9b Phosphorylation

**DOI:** 10.1002/advs.78023

**Published:** 2026-09-27

**Authors:** Lixiang Xie, Ziye Huang, Zhiyuan Zhang, Yiqiang Zhu, Xiaoqing Liu, Lei Wang, Tongyu Bi, Taizhen Liang, Jintao Lai, Meilin Hu, Guochang Qiu, Shiqi Xiao, Sen Liu, Yaoming Liu, Haiyue Rao, Tao Chen, Haojie Peng, Bin Zhang, Jia Tang, Qianying Li, Yaxin Li, Yuxin Hou, Weibo Yang, Hewei Jiang, Xiancai Ma

**Affiliations:** ^1^ Institute of Human Virology Zhongshan School of Medicine Sun Yat‐sen University Guangzhou China; ^2^ Guangzhou National Laboratory Guangzhou International Bio‐Island Guangzhou China; ^3^ State Key Laboratory of Respiratory Disease National Clinical Research Center for Respiratory Disease Guangzhou Institute of Respiratory Health The First Affiliated Hospital of Guangzhou Medical University Guangzhou China; ^4^ Shanghai Center for Systems Biomedicine Key Laboratory of Systems Biomedicine (Ministry of Education) Shanghai Jiao Tong University Shanghai China; ^5^ State Key Laboratory of Drug Research Shanghai Institute of Materia Medica Chinese Academy of Sciences Shanghai China; ^6^ University of Chinese Academy of Sciences Beijing China; ^7^ School of Biology and Biological Engineering South China University of Technology Guangzhou China; ^8^ College of Life Science and Technology Huazhong University of Science and Technology Wuhan China; ^9^ Department of Microbiology Li Ka Shing Faculty of Medicine The University of Hong Kong Pokfulam Hong Kong SAR China; ^10^ Lingang National Laboratory Shanghai China

**Keywords:** innate immunity, ORF9b, PPM1A, *Sarbecovirus*, STAT2

## Abstract

Coronaviruses have persistently triggered global pandemics in the 21^st^ century, featuring either high transmissibility or high pathogenicity. A hallmark of these infections is the delayed activation of innate immune responses, resulting in dysregulated antiviral signaling and uncontrolled viral replication. Multiple viral proteins and hijacked host proteins contribute to immune evasion, representing potential therapeutic targets. Here, we identify viral ORF9b as a conserved accessory protein across the *Sarbecovirus* subgenus that consistently suppresses innate immune responses by recruiting the protein phosphatase, Mg^2+^/Mn^2+^‐dependent 1A (PPM1A). Mechanistically, PPM1A exerts dual roles by directly dephosphorylating ORF9b and indirectly downregulating STAT2 phosphorylation, thereby suppressing RIG‐I/MAVS and JAK‐STAT signaling pathways. The PPM1A inhibitor SMIP‐031 inhibits coronavirus replication and restores the antiviral innate immune homeostasis. These findings reveal a conserved immune‐evasion strategy in sarbecoviruses and highlight the ORF9b‐PPM1A axis as a potential target for broad‐spectrum sarbecovirus therapeutics to help prevent future pandemics.

## Graphical abstract

Sarbecovirus ORF9b recruits host phosphatase PPM1A to suppress innate immunity through dual mechanisms. PPM1A directly dephosphorylates ORF9b to sustain its activity and indirectly downregulates STAT2 phosphorylation to impair interferon signaling. Pharmacological inhibition of PPM1A restores antiviral responses and limits sarbecovirus replication, revealing a host‐directed antiviral strategy. More details can be found in the Research Article by Xiancai Ma and co‐workers.

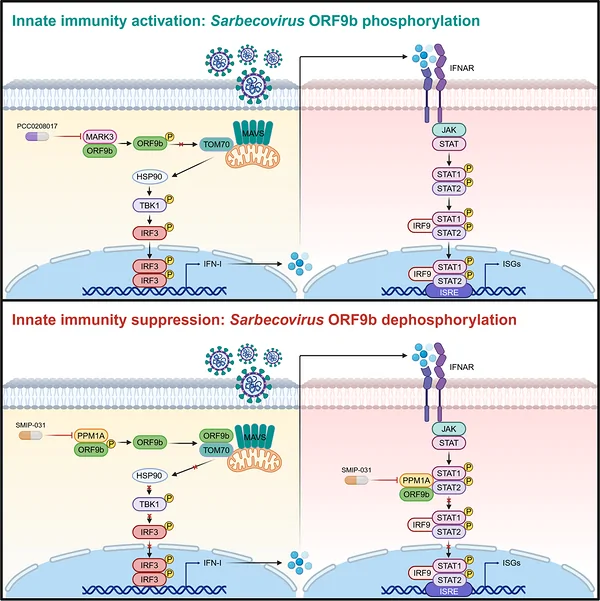

## 1 Introduction

Coronaviruses (CoVs) are enveloped positive‐sense single‐stranded RNA viruses, belonging to the order *Nidovirales*, family *Coronaviridae*, and subfamily *Orthocoronavirinae*, which encompass *Alpha‐*, *Beta‐*, *Gamma‐*, and *Deltacoronavirus* genera [1]. To date, seven human coronaviruses, including HCoV‐NL63, HCoV‐229E, HCoV‐OC43, HCoV‐HKU1, SARS‐CoV, MERS‐CoV, and SARS‐CoV‐2, have been identified. All of these human coronaviruses belong to *Alphacoronavirus* or *Betacoronavirus*, and are believed to have originated from zoonotic spillover events [2, 3]. SARS‐CoV, MERS‐CoV, and SARS‐CoV‐2 have caused pandemics of severe respiratory disease, whereas the remaining four coronaviruses are generally associated with mild respiratory symptoms [4]. In addition, two other zoonotic coronaviruses, Hu‐PDCoV and CCoV‐HuPn‐2018, have occasionally been detected in humans with limited transmission [5, 6]. Accumulating evidence suggests that bats, civets, pangolins, and camels serve as potential natural or intermediate hosts of human coronaviruses, highlighting the critical role of cross‐species transmission in coronavirus emergence and evolution [7].

Innate immunity constitutes the first line of the host defense against invading pathogens. However, during coronavirus infection, activation of innate immune responses is markedly delayed, resulting in impaired interferon responses, uncontrolled viral replication, and systemic immune dysregulation [8]. Notably, the extent of innate immune suppression strongly correlates with clinical prognosis, underscoring its pathogenic significance. Nearly all coronavirus‐encoded proteins, particularly those of SARS‐CoV‐2, have been reported to antagonize the innate immune signaling [9]. Coronavirus non‐structural proteins NSP13, NSP14, and NSP16 cooperatively modify the 5'‐ppp cap of viral RNA to mimic host mRNA, thereby evading retinoic acid‐inducible gene I (RIG‐I) recognition [10]. In parallel, the coronavirus nucleocapsid (N), membrane (M), and NSP5 proteins directly disrupt both the RIG‐I/mitochondrial antiviral‐signaling protein (MAVS)‐TANK‐binding kinase 1 (TBK1)‐interferon regulatory factor 3 (IRF3) pathway and the Janus kinase‐signal transducer and activator of transcription (JAK‐STAT) signaling pathway, resulting in reduced TBK1 phosphorylation, impaired IRF3 nuclear translocation, and decreased signal transducer and activator of transcription 1/2 (STAT1/STAT2) phosphorylation [11, 12, 13]. Moreover, papain‐like protease (PL^pro^) and 3C‐like protease (3CL^pro^/M^pro^) antagonize innate immunity by cleaving or deubiquitinating key signaling molecules [14, 15].

In addition to structural and non‐structural proteins, coronavirus‐encoded accessory proteins are also extensively involved in immune evasion. The accessory protein ORF9b has emerged as a potent innate immune antagonist. Both our work and previous studies have demonstrated that ORF9b proteins of SARS‐CoV and SARS‐CoV‐2 efficiently suppress type I interferon (IFN‐I) production [16, 17]. Proteomic and immunoblot analyses further indicate that ORF9b protein levels increase rapidly following SARS‐CoV‐2 infection [18, 19, 20]. Notably, multiple variants of concern (VOCs), including SARS‐CoV‐2 Alpha and Omicron lineages, exhibit significantly elevated ORF9b sgRNA levels, raising the possibility that enhanced ORF9b expression may contribute to improved immune evasion [18, 20, 21]. ORF9b has also been detected in virions and elicits antibody responses in infected individuals, indicating that ORF9b is a virion‐associated protein with immunogenicity during natural infection [22, 23, 24, 25]. Mechanistically, ORF9b localizes to mitochondria and interacts with the translocase of outer mitochondrial membrane 70 (TOM70), thereby disrupting the recruitment of the heat shock protein 90‐TBK1‐IRF3 (HSP90‐TBK1‐IRF3) complex and attenuating downstream interferon signaling [26, 27, 28]. Structural studies have revealed that ORF9b undergoes conformational rearrangement upon binding to TOM70, which remodels the interaction interface and displaces key residues such as R192 that are required for HSP90 association [29, 30, 31]. Beyond TOM70, ORF9b interferes with MAVS stability, NF‐κB essential modulator (NEMO) ubiquitination, and TBK1 activation, thereby broadly suppressing type I and type III interferon responses [17, 20].

Post‐translational modifications (PTMs), particularly phosphorylation, play pivotal roles in coronavirus infection by dynamically regulating both host and viral proteins. Coronavirus infection triggers extensive remodeling of host kinase networks, resulting in widespread phosphorylation across cellular and viral proteins. In host cells, activation of innate immunity involves phosphorylation of key signaling molecules, such as MAVS, TBK1, IRF3, STAT1, and STAT2. Phosphoproteomic analyses of SARS‐CoV‐2‐infected cells have confirmed broad changes in global phosphorylation patterns, accompanied by the activation of kinases including casein kinase II (CK2) and p38 mitogen‐activated protein kinase (p38 MAPK) [18]. Among these signaling molecules, TBK1 functions as a central kinase that phosphorylates IRF3 and drives type I interferon production. The activation of TBK1 depends on its phosphorylation at Ser172 within the kinase activation loop [32]. Notably, this phosphorylation is reversed by protein phosphatase, Mg^2+^/Mn^2+^‐dependent 1A (PPM1A; also known as PP2Cα), which directly dephosphorylates TBK1 at Ser172, thereby attenuating STING‐TBK1‐IRF3 signaling [33]. PPM1A belongs to the PP2C family of metal‐dependent Ser/Thr phosphatases and requires millimolar Mg^2+^ or Mn^2+^ for catalysis, with a labile third metal ion at the Asp146/Asp239 subsite being essential for activity [34]. Structural studies have revealed that PPM1A accommodates structurally diverse phosphoprotein targets through a conformationally plastic Flap subdomain [35, 36]. Downstream of interferon production, STAT2 is a critical component of the JAK‐STAT pathway. Upon type I interferon stimulation, STAT2 is phosphorylated at Tyr690 by TYK2, enabling its heterodimerization with STAT1 and association with IRF9 to form the ISGF3 complex [37]. In addition to this canonical tyrosine phosphorylation, STAT2 contains multiple Ser/Thr residues that modulate its activity. Phosphorylation of Ser287 accelerates Tyr690 dephosphorylation and terminates signaling, while Thr404, phosphorylated by TBK1 and IKKε, regulates STAT2 intracellular localization [38, 39]. In parallel, coronavirus proteins themselves also undergo phosphorylation during infection. Phosphorylation of the N protein has been widely reported in multiple human‐ and animal‐derived coronaviruses [40, 41, 42, 43, 44]. Phosphorylation of the N protein enhances its oligomerization and modulates its subcellular localization, thereby facilitating the assembly of N and viral RNAs [45, 46, 47, 48]. Beyond N, proteomic analyses have identified phosphorylation events on other viral proteins, including spike (S), M, NSP1, NSP2, NSP3, ORF3a, and ORF9b in SARS‐CoV, as well as S, M, NSP2, NSP3, NSP6, NSP9, NSP14, ORF3a, and ORF9b in SARS‐CoV‐2, underscoring the broad regulatory role of phosphorylation in viral life cycles [18, 40, 41, 45, 49].

Notably, ORF9b phosphorylation has emerged as a key determinant of its immune‐antagonistic activity. Mass spectrometry analyses have identified multiple phosphorylation sites within ORF9b [18, 29, 40, 45, 49]. ORF9b binds TOM70 and interferes with the recruitment of the HSP90‐TBK1‐IRF3 complex. However, phosphorylation at S53 of ORF9b weakens its interaction with TOM70, leading to reduced binding affinity and diminished co‐localization with TOM70 [50]. Beyond phosphorylation, the stability and functional activities of ORF9b are dynamically regulated by the host ubiquitination machinery. Host E3 ubiquitin ligases, such as the Cullin‐RING ligase 5 (CRL5) complex, facilitate ORF9b ubiquitination and subsequent proteasomal degradation, whereas deubiquitinases, including ubiquitin‐specific protease 29 (USP29), counteract this process to stabilize ORF9b [51, 52, 53]. Collectively, these findings highlight that PTMs cooperatively fine‐tune ORF9b stability, subcellular localization, and immune‐antagonistic function, thereby shaping viral replication and host antiviral responses.

Although both SARS‐CoV and SARS‐CoV‐2 ORF9b proteins suppress type I interferon signaling, their mechanisms of innate immune antagonism are not identical, indicating functional divergence between homologs. Given the critical role of ORF9b in antiviral suppression and the high pathogenicity of these viruses, it remains unclear whether ORF9b homologs exist in other coronaviruses and whether they operate through a conserved immune‐evasion strategy. Determining the conserved mechanism is important, as a shared ORF9b‐dependent strategy could provide a common target for broad‐spectrum antiviral development. Currently, no clinically approved agents directly target ORF9b, and therapeutic approaches aimed at modulating its function remain limited. Elucidating the conservation, regulatory mechanisms, and functional diversity of ORF9b‐mediated immune antagonism could therefore provide a foundation for rational antiviral development.

In this study, we identified ORF9b as a conserved protein within the *Sarbecovirus* subgenus and addressed a critical knowledge gap by systematically investigating shared mechanisms underlying sarbecovirus ORF9b‐mediated immune evasion. We revealed that ORF9b proteins were uniquely present in *Sarbecovirus*, a member of the *Betacoronavirus* genus, including bat, civet, pangolin, and human coronaviruses. Functionally, all verified sarbecovirus ORF9b homologs suppressed innate immune responses. We identified the host protein phosphatase PPM1A as a critical interactor of sarbecovirus ORF9b homologs utilizing multiple proteomic analyses. The *PPM1A* expression was negatively correlated with *ISG15* levels while positively associated with the severity of COVID‐19. Both ORF9b and PPM1A enhanced replication of authentic SARS‐CoV and SARS‐CoV‐2. Mechanistically, ORF9b bound to STAT2 and recruited PPM1A to indirectly downregulate STAT2 phosphorylation, impairing STAT2 nuclear translocation and suppressing downstream ISG expression. Concurrently, PPM1A directly dephosphorylated ORF9b, promoting the disruption of the TOM70‐HSP90 complex and thereby attenuating TBK1‐IRF3 activation and IFN‐I production. Pharmacological inhibition of PPM1A with SMIP‐031 suppressed viral replication and restored innate immune responses both in vitro and in vivo. These findings not only establish dual mechanisms of ORF9b function across sarbecoviruses but also identify PPM1A as a potential therapeutic target. Our work provides a conceptual framework for developing PPM1A‐targeted interventions as broad‐spectrum antiviral strategies against current and future sarbecovirus outbreaks.

## 2 Results

### 2.1 ORF9b Is Evolutionarily Conserved across the *Sarbecovirus* Subgenus

**FIGURE 1:**
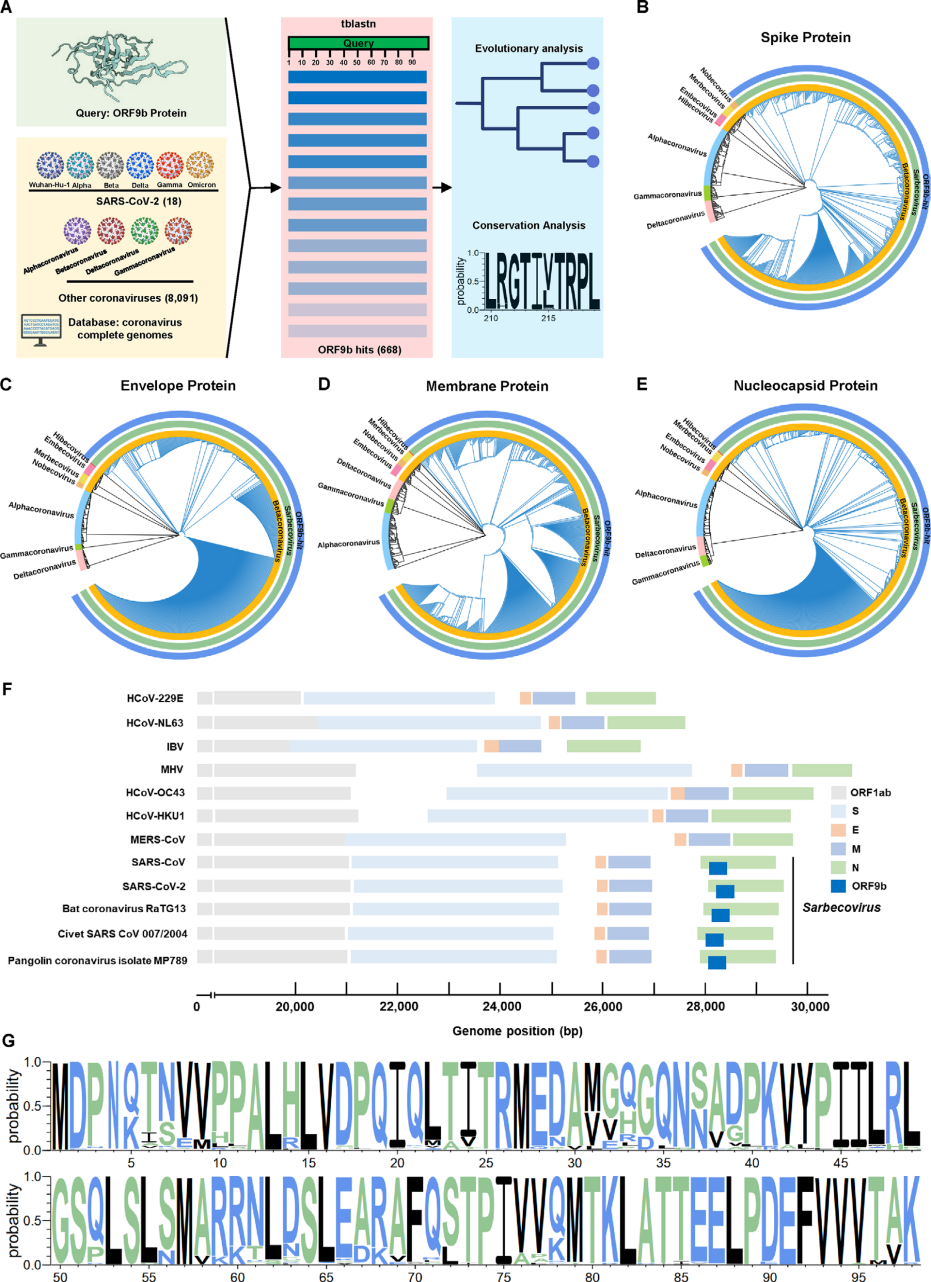
ORF9b is a lineage‐restricted and evolutionarily conserved accessory protein of *Sarbecovirus*. (A) Workflow of ORF9b identification and conservation analysis. The ORF9b sequence from SARS‐CoV‐2 (Wuhan‐Hu‐1) was used as a query to search a comprehensive coronavirus genome database using tblastn. A total of 668 ORF9b homologs were identified and subjected to phylogenetic and conservation analyses. Sequence conservation was illustrated by a sequence logo derived from aligned ORF9b homologs. (B‐E) Phylogenetic distribution of structural proteins across representative and ORF9b‐hit coronaviruses. Circular phylogenetic trees annotated with protein presence highlighted the lineage specificity of ORF9b compared with canonical structural proteins, including spike (S) (B), envelope (E) (C), membrane (M) (D), and nucleocapsid (N) (E). (F) Schematic representation of representative human and animal coronavirus genomes. Canonical genes (ORF1ab, S, E, M, and N) were indicated in gray, blue, orange, purple and green, respectively, with ORF9b uniquely marked in dark blue. (G) ORF9b‐hit amino acid sequences were aligned with MAFFT and the conservation analysis was performed with WebLogo 3.

Unlike the canonical structural proteins spike (S), envelope (E), membrane (M), and nucleocapsid (N), which are broadly conserved across coronaviruses, the evolutionary distribution of the accessory protein ORF9b has not been systematically defined [54]. Previous studies have reported the presence of ORF9b in both SARS‐CoV and SARS‐CoV‐2 [54]. To investigate the evolutionary distribution of ORF9b across coronaviruses, we first constructed a comprehensive coronavirus genome database. The database included complete genome sequences of SARS‐CoV‐2 original strains, SARS‐CoV‐2 variant isolates sourced from GISAID, and all other coronavirus genomes with a minimum length of 20 kb available from the NCBI Virus database. Using the SARS‐CoV‐2 (Wuhan‐Hu‐1) ORF9b protein sequence as a query, we performed tblastn searches against this database. The initial tblastn search returned 668 significant hits (E‐value < 1×10^−5^), with amino acid identity ranging from 40.2% to 100% and query coverage from 51.5% to 100% (Figure 1A; Figure S1A). Notably, 664 of the 668 hits (99.4%) showed query coverage ≥ 88%, and 630 of the 668 hits (94.3%) exhibited sequence identity ≥ 70%. Although genomes ≥ 20 kb were included in the initial database to avoid prematurely excluding divergent or incomplete coronavirus genomes, all ORF9b‐positive hits were derived from genomes ≥ 25.96 kb in length, with 98.95% exceeding 29 kb (661/668), consistent with full‐length coronavirus genomes (Table S1). To determine the phylogenetic context of ORF9b‐containing viruses, we next reconstructed evolutionary trees based on the corresponding structural proteins, including S, E, M, and N, from the hit‐bearing accessions along with representative coronavirus protein sequences spanning all four genera. Phylogenetic analyses of these canonical structural proteins revealed that all ORF9b‐positive viruses clustered within the *Sarbecovirus* subgenus. In contrast, no ORF9b homologs were detected in other coronavirus lineages, including Alpha‐, Beta‐ (non‐*Sarbecovirus*), Gamma‐, or Deltacoronaviruses under the same search criteria, despite the presence of conserved structural proteins (Figure 1B–E).

Consistent with the above lineage‐specific distribution, the genome organization analysis further demonstrated that the *ORF9b* gene was embedded within the N‐terminal region of the *N* gene exclusively in sarbecovirus genomes, whereas no significant homolog was identified in non‐sarbecovirus genomes under the same search criteria (Figure 1F). To characterize sequence conservation of ORF9b within sarbecoviruses, identified ORF9b protein sequences were aligned for sequence logo analysis. The resulting logo revealed a high degree of amino acid conservation across ORF9b sequences, suggesting that this protein is subject to strong evolutionary constraints (Figure 1G). We performed pairwise sequence similarity analyses at both the nucleotide and amino acid levels. ORF9b sequences from representative sarbecoviruses displayed high sequence identity, with particularly strong conservation among SARS‐CoV‐2 variants. SARS‐CoV‐2 and its variants shared high nucleotide and amino acid similarity with bat coronavirus RaTG13 and pangolin coronavirus MP789, whereas SARS‐CoV was more closely related to civet SARS CoV 007/2004, underscoring their zoonotic origin (Figure S1B). The multiple sequence alignment further revealed the extensive conservation across ORF9b proteins, including eight known phosphorylation sites (Y42, S50, S53, S63, T72, T83, T84, and T95), among which S50 and S53 have been shown to be essential for antagonizing host type I interferon signaling, with only limited variability at a few peripheral residues (Figure S1C). A few lineage‐specific substitutions were observed, such as the P10S mutation and the loss of the ENA/EDA motif in SARS‐CoV‐2 Omicron isolates compared with earlier circulating variants. Taken together, these data indicated that ORF9b proteins were highly conserved and subjected to functional constraints across sarbecoviruses, supporting the possibility that ORF9b represented a potential target for antiviral strategies aimed at both existing and newly emerging sarbecovirus strains.

### 2.2 Sarbecovirus ORF9b Functions as a Potent Antagonist of Innate Immune Responses

**FIGURE 2:**
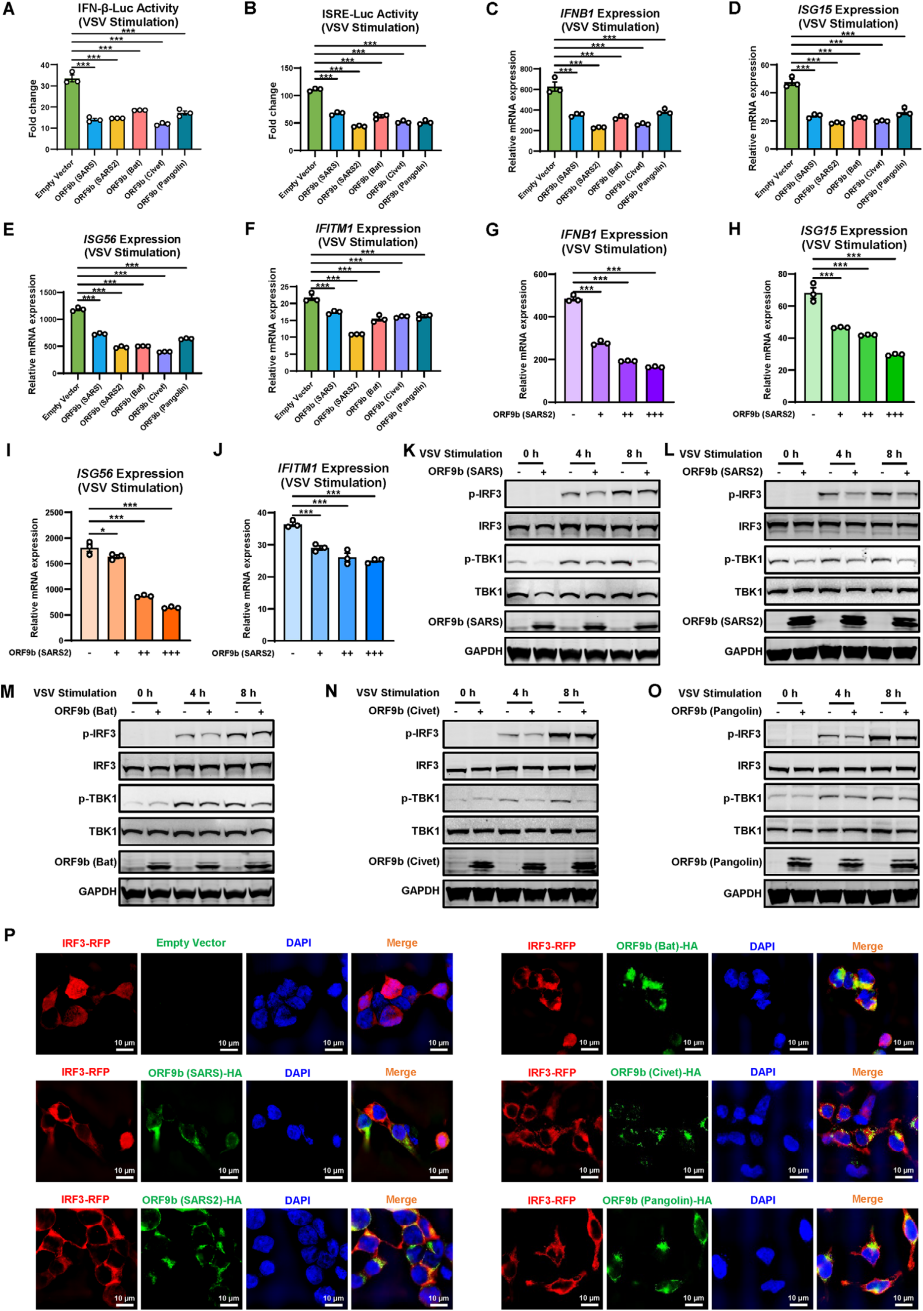
Sarbecovirus ORF9b acts as a suppressor of host innate immune responses. (A, B) ORF9b sequences from SARS‐CoV (Tor2), SARS‐CoV‐2 (Wuhan‐Hu‐1), bat coronavirus RaTG13, civet SARS CoV 007/2004, and pangolin coronavirus MP789 were codon‐optimized and synthesized to construct various ORF9b‐expressing plasmids, which were designated as ORF9b (SARS), ORF9b (SARS2), ORF9b (Bat), ORF9b (Civet), and ORF9b (Pangolin), respectively. HEK293T cells were transfected with indicated ORF9b‐expressing plasmids or the empty vector, together with IFN‐β‐Luc and pRL‐TK plasmids, or with ISRE‐Luc and pRL‐TK plasmids. At 24 hours post‐transfection (h.p.t.), cells were infected with VSV for 12 hours (h) prior to luciferase measurement. Fold changes of firefly luciferase activity were normalized to *Renilla* luciferase activity. (C‐F) RT‐qPCR analysis of mRNA expression levels of *IFNB1*, *ISG15*, *ISG56*, and *IFITM1* in HEK293T cells transfected with indicated plasmids for 24 h, followed by infection with VSV for 12 h. (G‐J) HEK293T cells were overexpressed with increasing amounts of ORF9b (SARS2) for 24 h, then infected with VSV for 12 h. RT‐qPCR analysis was performed as in (C‐F). (K‐O) A549 cells were overexpressed with ORF9b homologs for 24 h, followed by infection with VSV for the indicated time. Cells were harvested for western blot analysis with antibodies against ORF9b, total TBK1, total IRF3, phosphorylated TBK1 (p‐TBK1), and p‐IRF3. (P) HEK293T cells were co‐overexpressed with ORF9b homologs and IRF3‐RFP. At 24 h.p.t., cells were stimulated with poly(I:C) for 8 h, followed by immunofluorescence (IF) analysis to visualize IRF3 nuclear translocation. Scale bar, 10 µm. Data in (A‐J) were presented as mean ± SEM in biological triplicates. *P* values were calculated by one‐way ANOVA with Dunnett's multiple comparisons tests. *P < 0.05, ***P < 0.001.

ORF9b has been found to be rapidly expressed upon SARS‐CoV‐2 infection, regulates mitochondrial function, modulates host metabolism, and influences inflammatory signaling [55, 56, 57, 58]. Notably, the most extensively studied and best‐established function of ORF9b is its antagonism of innate immunity, particularly through suppression of type I and III interferon production by targeting the RIG‐I/MAVS signaling axis [17, 20]. To investigate whether sarbecovirus ORF9b universally antagonized host antiviral defenses, we generated expression constructs encoding ORF9b from human, bat, pangolin and civet coronavirus isolates. We first examined their effects on the activation of interferon‐β (IFN‐β) and interferon‐stimulated response element (ISRE) promoters using dual‐luciferase reporter assays. The results showed that the overexpression of various sarbecovirus ORF9b significantly suppressed the activation of both promoters upon vesicular stomatitis virus (VSV) infection, indicating that sarbecovirus ORF9b might interfere with multiple stages of innate immune signaling cascades (Figure 2A,B). To further characterize the interferon‐antagonistic activity, we assessed the gene expression of type I interferon and interferon‐stimulated genes (ISGs) upon VSV infection. All ORF9b homologs consistently reduced the induction of *IFNB1*, *ISG15*, *ISG56*, and interferon‐induced transmembrane protein 1 (*IFITM1*) (Figure 2C–F). Notably, the inhibitory effects of SARS‐CoV‐2 ORF9b displayed dose‐dependent patterns (Figure 2G–J).

To assess whether ORF9b‐mediated immune suppression was stimulus‐dependent, we examined *IFNB1* and ISG expression upon infection with VSV or Sendai virus (SeV), or stimulation with polyinosinic:polycytidylic acid [poly(I:C)]. Under all these conditions, the presence of ORF9b consistently attenuated the induction of *IFNB1*, *ISG15*, and *ISG56* (Figure S2A‐I). Furthermore, ORF9b derived from other bat‐origin sarbecoviruses also significantly suppressed the expression of these antiviral genes, supporting a conserved immune‐antagonistic function across viral origins (Figure S2J–L). Given that SARS‐CoV‐2 Omicron variants harbor unique deletions or mutations in ORF9b, we further examined the impact of these changes on the innate immune antagonism. Western blot analysis confirmed that all ORF9b constructs were expressed at comparable protein levels upon transfection (Figure S2M–O). ORF9b with P10S substitution, ENA/EDA deletion, or derived from the Omicron JN.1 isolate (harboring both P10S substitution and ENA/EDA deletion) retained comparable inhibitory capacities on IFN‐β and ISG expression relative to the wild‐type ORF9b (Wuhan‐Hu‐1). These results indicated that despite sequence divergence, animal coronavirus‐ and SARS‐CoV‐2 Omicron‐derived ORF9b maintained robust interferon‐antagonistic activity.

The antiviral signal transduction of innate immunity relies on the phosphorylation of multiple signaling molecules, including TBK1, IRF3, and many others, resulting in the corresponding downstream changes in IFN and ISG transcription [59, 60, 61, 62, 63]. Having established that ORF9b potently suppressed downstream IFN and ISG expression, we noted that mRNA readouts reflected the combined output of IFN induction and IFN‐mediated signaling. To distinguish between these two layers, we next examined the phosphorylation of TBK1 and IRF3, which are key upstream signaling events specifically reporting on the RIG‐I/MAVS‐TBK1‐IRF3 axis of interferon induction. We found that sarbecovirus ORF9b homologs attenuated the phosphorylation of both TBK1 and IRF3 to varying extents following VSV infection (Figure 2K–O). Since IRF3 phosphorylation is required for its dimerization and nuclear translocation, we co‐transfected cells with sarbecovirus ORF9b‐ and IRF3‐RFP‐expressing plasmids. The results showed that all ORF9b homologs impaired the poly(I:C)‐induced nuclear translocation of IRF3 (Figure 2P). Altogether, these results demonstrated that sarbecovirus ORF9b acted as a conserved and potent antagonist of innate immune responses, suppressing type I interferon production and ISG expression across diverse stimuli and viral origins.

### 2.3 The ORF9b‐STAT2 Axis Interferes with the JAK‐STAT Signaling Pathway

**FIGURE 3:**
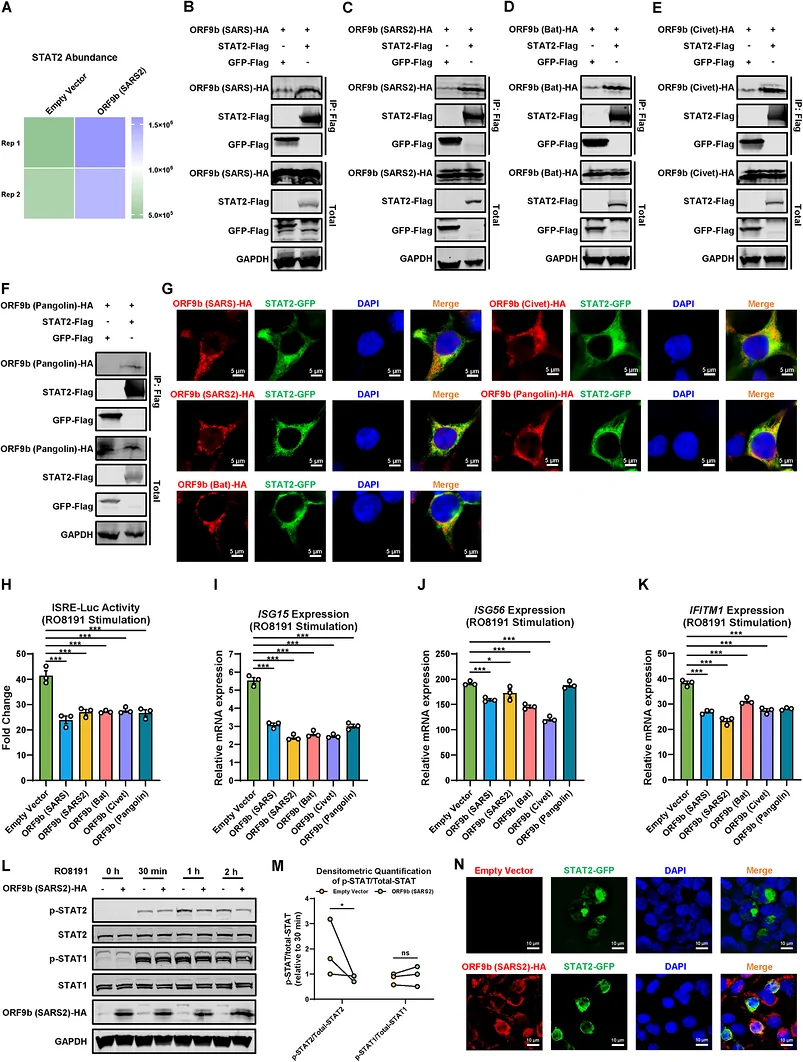
ORF9b binds STAT2 and suppresses JAK‐STAT signaling. (A) STAT2 abundance in ORF9b (SARS2) and empty vector groups in the Co‐IP‐MS dataset. (B‐F) Anti‐Flag Co‐IP assays with Flag‐tagged STAT2 and HA‐tagged ORF9b homologs, including ORF9b (SARS) (B), ORF9b (SARS2) (C), ORF9b (Bat) (D), ORF9b (Civet) (E), ORF9b (Pangolin) (F). (G) STAT2‐GFP was co‐overexpressed with HA‐tagged ORF9b homologs for IF analysis. Scale bar, 5 µm. (H) ISRE‐Luc reporter activity in HEK293T cells overexpressing indicated ORF9b homologs and stimulated with RO8191. (I‐K) The mRNA expression levels of *ISG15* (I), *ISG56* (J), and *IFITM1* (K) in HEK293T cells that overexpressed with sarbecovirus ORF9b homologs and stimulated with RO8191. (L) A549 cells were overexpressed with ORF9b (SARS2) for 24 h, followed by stimulation with RO8191 for indicated times. Western blot analysis was performed with antibodies against total STAT1, total STAT2, p‐STAT1, and p‐STAT2. (M) Densitometric quantification of p‐STAT2/total‐STAT2 (left) and p‐STAT1/total‐STAT1 (right) from blots shown in (L). Each dot represented one time point (30 min, 1 h, and 2 h post‐RO8191 stimulation). Empty vector and ORF9b (SARS2) groups were compared. (N) STAT2‐GFP and ORF9b (SARS2)‐HA were co‐overexpressed in HEK293T cells, followed by stimulation with RO8191 for 8 h. Nuclear translocation of STAT2 was analyzed by fluorescence microscopy. Scale bar, 10 µm. *P* values in (H‐K) were calculated by one‐way ANOVA with Dunnett's multiple comparisons tests, while *P* values in (M) were calculated by two‐way ANOVA with Šidák's multiple comparisons tests. ns, not significant. *P < 0.05, ***P < 0.001.

Our above results have demonstrated that sarbecovirus ORF9b attenuated mRNA expression and phosphorylation levels of critical antiviral signaling components. Therefore, we investigated whether ORF9b interacted with key components of the innate immune signaling pathway. However, Co‐immunoprecipitation (Co‐IP) assays revealed that no interactions were detected between ORF9b and RIG‐I, MDA5, MAVS, TBK1, or IRF3, suggesting that ORF9b might modulate the phosphorylation of these molecules through an indirect mechanism (Figure S3A–E). To systematically identify host factors associated with ORF9b, we performed Co‐IP coupled with mass spectrometry (Co‐IP‐MS), which identified STAT2 as a potential ORF9b‐interacting candidate (Figure 3A). STAT2 is a central mediator of the downstream JAK‐STAT signaling of antiviral innate immunity [64]. The phosphorylated STAT2, together with phosphorylated STAT1 and IRF9, binds to ISRE promoters and activates the expression of ISGs. Our Co‐IP and immunofluorescence (IF) assays confirmed that sarbecovirus ORF9b homologs commonly interacted with STAT2 (Figure 3B–G). Further functional studies demonstrated that sarbecovirus ORF9b significantly suppressed the activation of ISRE promoters in response to the stimulation with RO8191, an agonist targeting interferon‐α/β receptor 2 (IFNAR2) that directly activates the JAK‐STAT pathway independently of the upstream RIG‐I/MAVS cascade (Figure 3H). Consistent with this observation, the overexpression of sarbecovirus ORF9b attenuated the induction of ISGs, including *ISG15*, *ISG56*, and *IFITM1* upon RO8191 stimulation compared to empty vector controls (Figure 3I–K). Mechanistically, STAT2 exerts its antiviral function through JAK‐mediated phosphorylation of both STAT1 and STAT2, enabling STAT1‐STAT2 dimerization and the formation of the interferon‐stimulated gene factor 3 (ISGF3) complex with IRF9 [65]. Consequently, the nuclear‐translocated ISGF3 complex activates the ISRE‐driven transcription of antiviral ISGs. Therefore, we examined whether the attenuated induction of ISGs was attributable to impaired STAT phosphorylation, a key upstream event in the JAK‐STAT signaling cascade. Strikingly, ORF9b selectively impaired STAT2 phosphorylation upon RO8191 stimulation, without affecting STAT1 phosphorylation (Figure 3L,M, and Figure S3F). The overexpression of ORF9b also impeded the nuclear translocation of STAT2 (Figure 3N). These data demonstrated that ORF9b potentially inhibited downstream innate immune signaling by targeting STAT2.

### 2.4 Sarbecovirus ORF9b Directly Binds to the Host Phosphatase PPM1A

**FIGURE 4:**
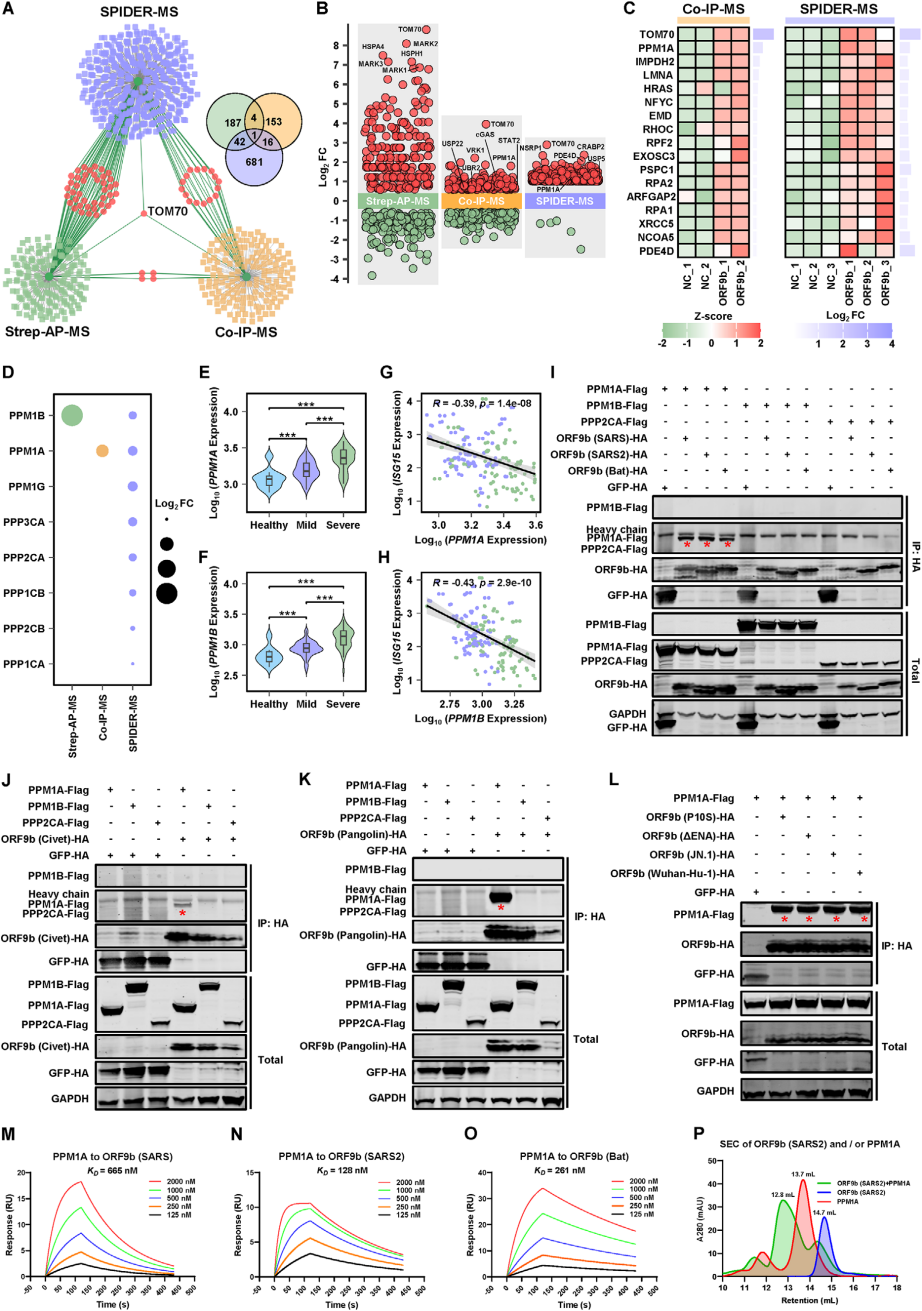
Proteomic identification and biochemical validation of PPM1A as an ORF9b‐interacting phosphatase. (A) Venn diagram and network representation showed the overlap of ORF9b‐enriched proteins across three proteomic datasets. Protein analysis was performed using SAINTexpress. Fold change thresholds were set at ≥ 1.5 for Strep‐AP‐MS and Co‐IP‐MS, and ≥ 2 for SPIDER‐MS. (B) Volcano plot showed differential protein enrichment identified from three proteomic datasets, including one publicly available dataset and two datasets generated in this study. The y‐axis represents log2 fold change. Red dots indicate enriched proteins (fold change ≥ 1.5 for Strep‐AP‐MS and Co‐IP‐MS, and ≥ 2 for SPIDER‐MS), whereas green dots represent depleted proteins. (C) Heatmaps showing enrichment (Z‐score) of overlapping proteins in Co‐IP‐MS and SPIDER‐MS datasets. Each column represents a biological replicate, and each row represents a protein. Bar charts depict the log2 fold change of selected candidates. (D) Bubble plot comparing the enrichment of the phosphatase family members across the three proteomic datasets. Bubble size represents log2 fold change. (E, F) Expression of *PPM1A* and *PPM1B* in clinical samples. Violin plots depicted relative mRNA expression levels in healthy, mild, and severe COVID‐19 individuals. (G‐H) Correlation analysis between *PPM1A* and *PPM1B* expression and *ISG15* expression in patient samples. Linear regression analysis was performed to evaluate the association between *ISG15* and *PPM1A*, or *PPM1B*. Pearson correlation coefficients (*R*) and corresponding two‐sided *P* values were calculated. (I) Co‐IP examination of interactions between ORF9b homologs with PPM1A, PPM1B, and PPP2CA. (J–L) Co‐IP examination of interactions between ORF9b and candidate phosphatases across different sarbecovirus ORF9b homologs and variants. (M‐O) Surface plasmon resonance (SPR) analysis of direct binding between recombinant PPM1A and ORF9b homologs. ORF9b (SARS) (M), ORF9b (SARS2) (N), and ORF9b (Bat) (O) were immobilized on the CM5 sensor chip, respectively, while PPM1A proteins were utilized as analytes. Sensorgrams and calculated binding constants (*K_D_*) were shown. (P) Size‐exclusion chromatography (SEC) of recombinant ORF9b and PPM1A proteins. Co‐incubation of both proteins resulted in an earlier elution peak compared with individual proteins. *P* values in (E‐F) were calculated using Kruskal‐Wallis tests followed by Dunn's post hoc comparisons tests. ***P < 0.001.

Given that ORF9b selectively downregulated STAT2 phosphorylation without affecting STAT1 phosphorylation, and that ORF9b itself lacks intrinsic phosphatase activity, we reasoned that ORF9b might recruit a host phosphatase to specifically downregulate STAT2 phosphorylation and thereby extinguish JAK‐STAT signaling. To search for such a phosphatase, we applied three proteomic approaches, including Co‐IP‐MS and spatial proximity‐induced direct enzymatic reporter (SPIDER)‐based mass spectrometry (SPIDER‐MS) generated in this study, and a publicly available Strep‑tag affinity purification mass spectrometry (Strep‑AP‑MS) (Figure S4A,B) [66]. Gene set enrichment analysis (GSEA) using the Reactome pathway database revealed that SARS‐CoV‐ and SARS‐CoV‐2‐related pathways were significantly enriched in the ORF9b‐associated protein set, including pathways involved in SARS‐CoV/SARS‐CoV‐2 infection, SARS‐CoV/SARS‐CoV‐2‐host interactions, and modulation of innate immune responses, indicating that ORF9b broadly recruited host proteins to modulate viral infection and host responses (Figure S4C). Comparative analysis revealed 17 common enriched proteins shared between Co‐IP‐MS and SPIDER‐MS, 43 shared between SPIDER‐MS and Strep‐AP‐MS, and 5 shared between Co‐IP‐MS and Strep‐AP‐MS (Figure 4A). Notably, TOM70 was consistently identified across all three independent proteomic approaches (Figure 4A,B). TOM70 is a mitochondrial adaptor protein previously reported to interact with ORF9b, validating the robustness of our screening strategy [26, 31, 67]. Protein‐protein interaction (PPI) network analysis revealed strong interactions among ORF9b‐interacting proteins, suggesting cooperative role in supporting ORF9b‐mediated functions (Figure S4D). The Strep‐AP‐MS dataset identified multiple kinases, including microtubule affinity‐regulating kinase 1 (MARK1), MARK2, and MARK3. Co‐IP‐MS and SPIDER‐MS captured several innate immune signaling molecules and host factors involved in post‐translational modification pathways, such as cyclic GMP‐AMP synthase (cGAS), STAT2, deubiquitinases ubiquitin‐specific protease 22 (USP22) and ubiquitin‐specific protease 5 (USP5), and the ubiquitin protein ligase E3 component N‐recognin 2 (UBR2) (Figure 4B). Interestingly, ORF9b interacted not only with kinases but also with phosphatases, implying that ORF9b could modulate host phosphorylation networks to fine‐tune immune signaling. In the overlap of enriched proteins identified by Co‐IP‐MS and SPIDER‐MS, both TOM70 and phosphatase PPM1A were present (Figure 4C). Extending our survey to phosphatases across all three datasets, we identified several ORF9b‐interacting phosphatases, including the PPM‐family phosphatases PPM1A, PPM1B, and PPM1G, together with catalytic subunits of PPP‐family phosphatases, namely PPP3CA, PPP2CA, PPP2CB, PPP1CA, and PPP1CB (Figure 4D). PPP1CA and PPP1CB are alternative catalytic subunit isoforms of PP1, whereas PPP2CA and PPP2CB are alternative catalytic subunit isoforms of PP2A. Among these proteins, *PPM1A*, *PPM1B*, and *PPP2CA* were significantly upregulated in mild cases of coronavirus disease 2019 (COVID‐19) and further elevated in severe cases compared with healthy individuals, exhibiting a stepwise increase with disease progression (Figure 4E,F and Figure S4E). In contrast, *PPM1G* and *PPP1CA* were negatively correlated with disease severity (Figure S4F,G). *PPP3CA* was only significantly upregulated in severe COVID‐19, with no difference between healthy individuals and mild cases (Figure S4H). Correlation analysis further demonstrated that *ISG15* was negatively correlated with the expression levels of *PPM1A*, *PPM1B*, *PPP2CA*, and *PPP3CA*, while positively correlated with the expression levels of *PPM1G* and *PPP1CA*, highlighting expression‐level heterogeneity among these proteins during disease progression (Figure 4G,H and Figure S4I–L). Given that phosphatases upregulated during infection are more likely to be exploited by the virus for immune evasion, we prioritized PPM1A, PPM1B, and PPP2CA for further validation.

As a positive control, ORF9b markedly bound to TOM70 (Figure S5A). The Co‐IP assays revealed a consistent interaction between PPM1A and all ORF9b homologs, as well as SARS‐CoV‐2 Omicron‐derived mutants (Figure 4I–L and Figure S5B). However, no detectable interaction was observed between ORF9b and PPM1B or PPP2CA, confirming the specificity of the ORF9b‐PPM1A association (Figure 4I–K). Furthermore, IF assays confirmed that TOM70 and PPM1A co‐localized with sarbecovirus ORF9b homologs, respectively, supporting a conserved interaction between sarbecovirus ORF9b and cellular PPM1A (Figure S5C,D). To verify the direct binding of PPM1A to ORF9b homologs, we performed surface plasmon resonance (SPR) assays utilizing in vitro purified proteins. Recombinant PPM1A proteins showed the highest binding affinity to SARS‐CoV‐2 ORF9b, with a binding constant of 128 nm, while PPM1A bound to SARS‐CoV ORF9b and bat coronavirus‐derived ORF9b with binding constants of 665 and 261 nm, respectively (Figure 4M–O). Consistently, the co‐incubation of recombinant SARS‐CoV‐2 ORF9b and PPM1A proteins followed by size‐exclusion chromatography (SEC) revealed an earlier elution peak compared with individual proteins, indicating the formation of ORF9b‐PPM1A protein complex (Figure 4P). Previous reports have shown that PPM1A interacts with short phosphopeptide substrates with binding affinities in the low micromolar range of 8.9 µm for a cyclic phosphopeptide [36]. The SPR‐measured binding constants of ORF9b for PPM1A were substantially tighter than these reported values, raising the possibility that ORF9b may serve either as a bona fide substrate that engages additional docking contacts beyond the catalytic interface, or as a competitive inhibitor that occupies the substrate recognition surface. Collectively, our above investigations revealed that sarbecovirus ORF9b homologs directly interacted with the cellular phosphatase PPM1A.

### 2.5 Structural Modeling and Mutational Validation of the ORF9b‐PPM1A Interface

**FIGURE 5:**
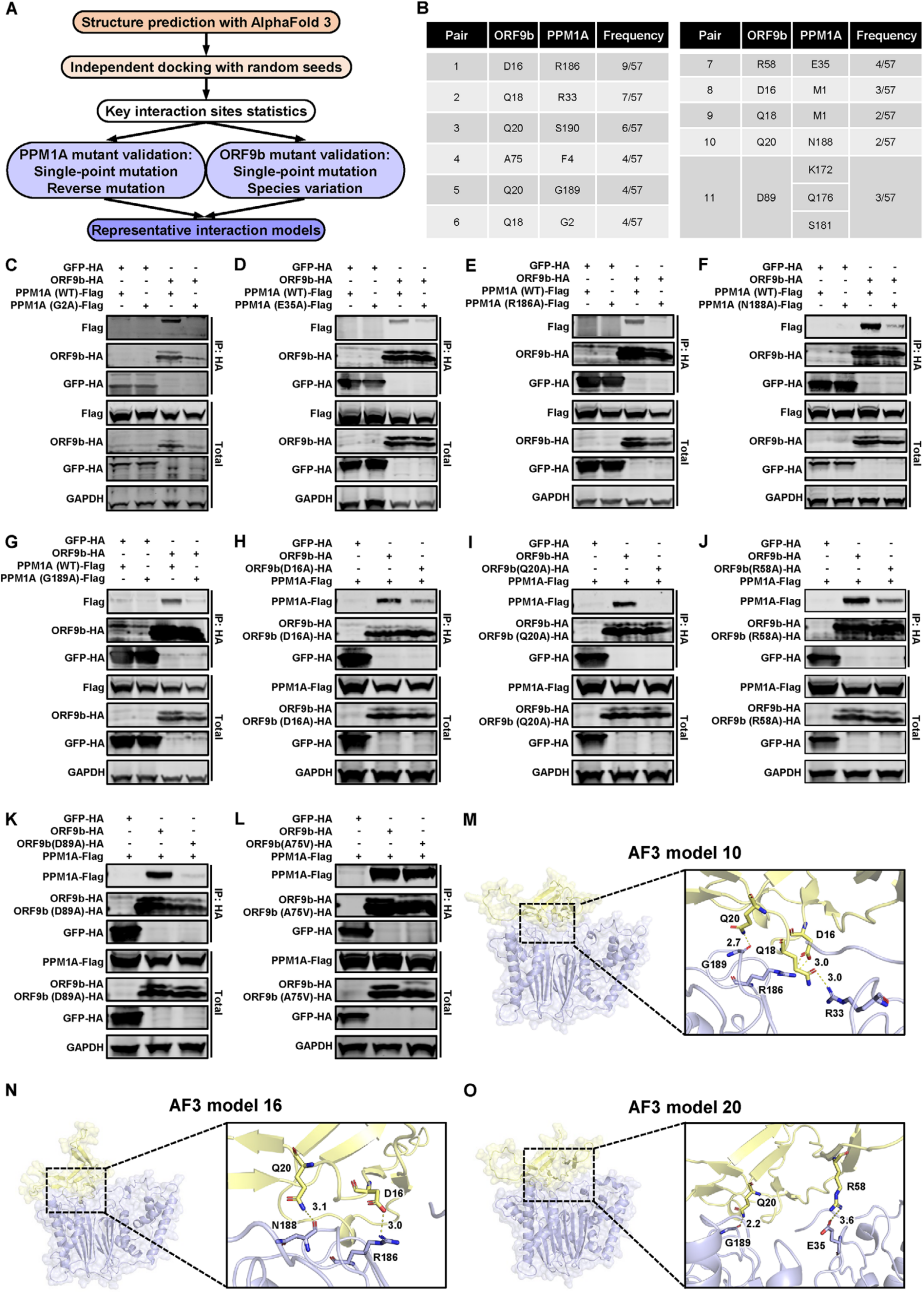
Structure‐guided identification of the ORF9b‐PPM1A interface. (A) Schematic workflow of structure‐guided interface mapping and validation. The structures of ORF9b and PPM1A were predicted and independently docked with AlphaFold 3 (AF3) using different random seeds. Key interaction sites were visualized in PyMOL, followed by point mutation and reverse mutation to validate their interactions. (B) Frequency analysis of predicted interfacial residue pairs across independent AF3 models. Residue pairs observed repeatedly among predicted complexes were listed with occurrence frequency, highlighting recurrent contacts between ORF9b and PPM1A. (C–G) Co‐IP assays validating key PPM1A interface residues. Flag‐tagged wild‐type (WT) PPM1A or its mutants (G2A, E35A, R186A, N188A, and G189A) were co‐overexpressed with HA‐tagged ORF9b, followed by anti‐HA immunoprecipitation and immunoblotting. (H–L) Co‐IP validation of ORF9b interface residues. WT ORF9b or its mutants (D16A, Q20A, R58A, D89A, and A75V) were verified for interaction with PPM1A. (M‐O) Representative AF3 structural models (models 10, 16, and 20) of the ORF9b‐PPM1A complex. Distances (Å) between interacting residues were indicated.

Having confirmed the direct and specific interaction between ORF9b and PPM1A, we initially aimed to determine the structure of the ORF9b‐PPM1A complex using X‐ray crystallography. However, all attempts failed because we were unable to obtain diffracting crystals under various conditions. We therefore employed AlphaFold 3 (AF3) to predict the structure of the ORF9b‐PPM1A complex, followed by point mutation and reverse mutation to validate their interactions (Figure 5A). Twenty predicted models were analyzed using PyMOL, revealing a total of 57 residue–residue contacts within a 4 Å distance. Multiple recurrent interaction hotspots were identified across predicted models, including the aspartic acid residue (D) at position 16 of ORF9b to the arginine residue (R) at position 186 of PPM1A (D16‐R186), Q18‐R33, Q20‐S190, A75‐F4, Q20‐G189, Q18‐G2, and R58‐E35 (Figure 5B). To assess the functional relevance of these predicted interfaces, we generated single‐site alanine residue substitution mutants on either ORF9b or PPM1A at the specified paired residues and examined their interactions by Co‐IP assays. Additional frequently appeared interaction hotspots including D89 on ORF9b and N188 on PPM1A were also verified. Co‐IP analysis revealed that the alanine residue substitution at G2 (G2A), E35A, R186A, N188A, and G189A on PPM1A, as well as D16A, Q20A, R58A, and D89A on ORF9b, markedly impaired the ORF9b‐PPM1A interaction, indicating that these residues were critical for the complex formation (Figure 5C–K). In contrast, F4A, R33A, and S190A on PPM1A, or Q18A on ORF9b, did not markedly impact the interaction (Figure S6A–D). Notably, the residue at position 75 of ORF9b is evolutionarily variable among sarbecoviruses, with either alanine or valine residue observed at this site (such as V75 in SARS‐CoV and A75 in SARS‐CoV‐2), suggesting potential functional tolerance or conservation of hydrophobic character at this interface. Consistently, the A75V mutation on ORF9b did not impair the interaction with PPM1A, indicating that this naturally occurring variation did not compromise PPM1A‐mediated regulation of ORF9b function (Figure 5L).

To further define the essential binding determinants, we generated a PPM1A mutant containing eight simultaneous alanine substitutions at predicted interface residues. Co‐IP assays showed that neither the eight‐site‐mutated PPM1A (8M) nor any of the corresponding single‐site revertant mutants were able to restore the interaction with ORF9b, suggesting that the binding complex of ORF9b‐PPM1A relied on conformational epitopes composed of multiple spatially adjacent residues rather than a single dominant contact (Figure S6E). Among the validated contact pairs, D16‐R186, Q20‐G189, Q20‐N188, and R58‐E35 were consistently observed across the 20 AF3 models and were simultaneously presented in three representative models (model 10, model 16, and model 20), supporting their functional importance in stabilizing the ORF9b‐PPM1A complex (Figure 5M–O). Notably, previous structural studies have established that both Arg33 and Arg186 of PPM1A participate in substrate recognition [36]. Our alanine‐scanning mutagenesis data revealed that only Arg186 was essential for ORF9b binding. This differential requirement indicated that ORF9b might engage PPM1A through a docking mode that shared the Arg186 anchoring point with canonical substrates but did not depend on Arg33, consistent with the conformational plasticity of the Flap subdomain [35].

### 2.6 PPM1A Is Hijacked by ORF9b to Downregulate STAT2 Phosphorylation and Subvert JAK‐STAT Immunity

**FIGURE 6:**
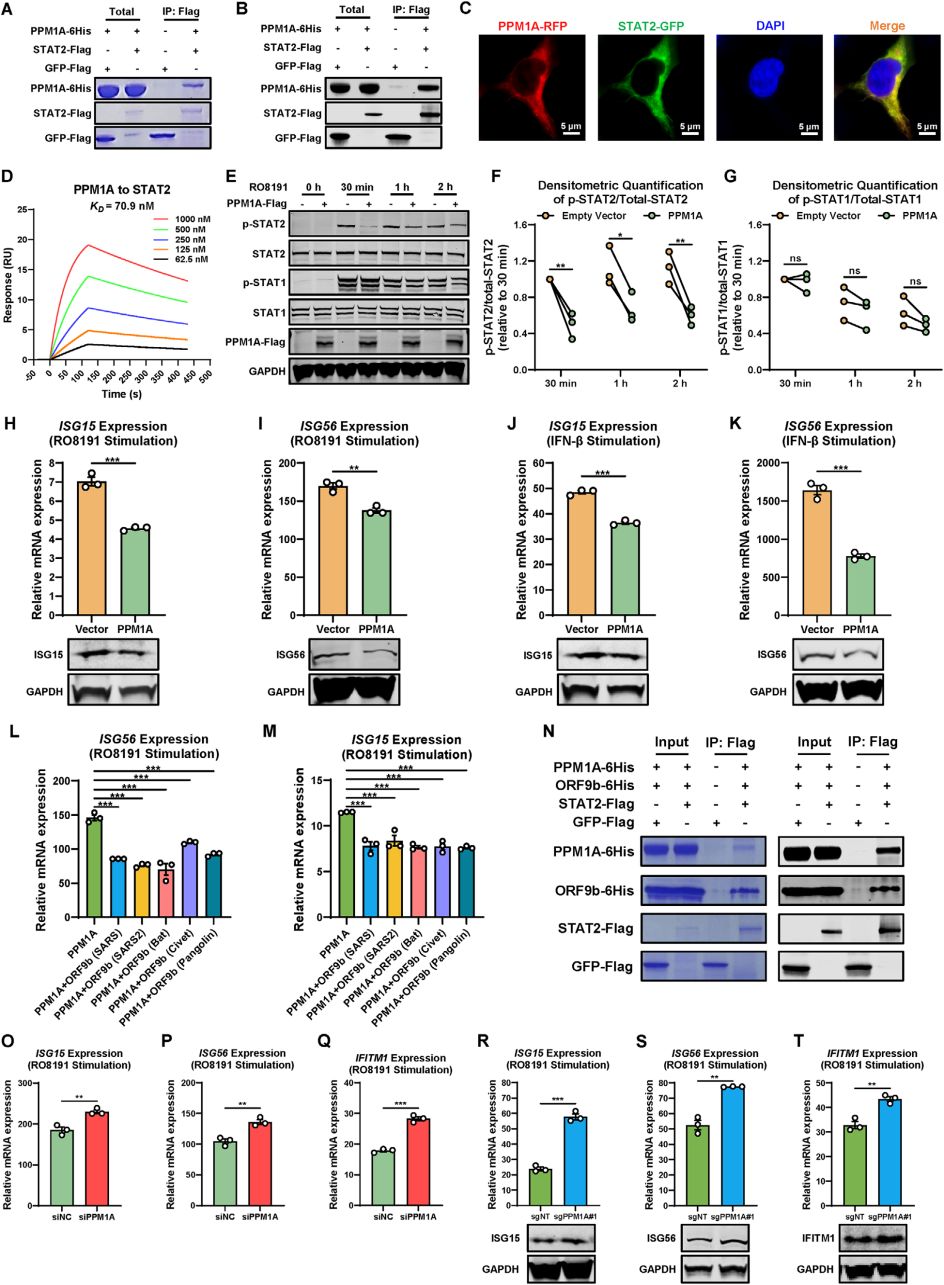
PPM1A downregulates STAT2 phosphorylation to mediate ORF9b‐induced JAK‐STAT suppression. (A, B) Anti‐Flag pull‐down assays were conducted using recombinant His‐tagged PPM1A and Flag‐tagged STAT2 proteins. Flag‐tagged GFP proteins served as negative controls. The interactions were assessed by Coomassie blue staining (A) and immunoblotting (B). (C) Co‐localization of STAT2‐GFP with PPM1A‐RFP in HEK293T cells. No RO8191 stimuli were added. Scale bar, 5 µm. (D) SPR assay of STAT2‐PPM1A binding affinity. Recombinant STAT2 proteins were immobilized on the CM5 sensor chip, with PPM1A utilized as analytes. (E) A549 cells were overexpressed with PPM1A for 24 h and stimulated with RO8191 for various time periods. Western blot analysis was conducted with antibodies against total STAT1, total STAT2, p‐STAT1, and p‐STAT2. (F, G) Densitometric quantification of p‐STAT2/total‐STAT2 (F) and p‐STAT1/total‐STAT1 (G) ratios from three independent biological replicates. p‐STAT2 and p‐STAT1 levels were normalized to total STAT2 and total STAT1, respectively, and expressed relative to the 30‐min time point within each replicate. (H, I) HEK293T cells were overexpressed with PPM1A or empty vector for 24 h, followed by stimulation with RO8191 for 12 h. *ISG15* (H) and *ISG56* (I) mRNA levels were determined by RT‐qPCR (top), and the corresponding protein levels were assessed by immunoblotting (bottom). (J, K) HEK293T cells were overexpressed with PPM1A or empty vector for 24 h, followed by stimulation with recombinant IFN‐β proteins for 12 h. *ISG15* (J) and *ISG56* (K) mRNA levels were determined by RT‐qPCR (top), and the corresponding protein levels were assessed by immunoblotting (bottom). (L, M) HEK293T cells were co‐overexpressed with PPM1A and various ORF9b homologs for 24 h. After stimulation with RO8191 for 12 h, the mRNA expression levels of *ISG15* (L) and *ISG56* (M) were quantified via RT‐qPCR. (N) Equal amounts (50 µg) of STAT2‐Flag or GFP‐Flag were incubated with PPM1A‐6His and ORF9b (SARS2)‐6His in the presence of anti‐Flag beads at 4°C overnight. The ternary complex formation was assessed by Coomassie staining (left) and western blot (right). (O‐Q) A549 cells were transfected with siRNAs targeting PPM1A or negative control (NC) for 12 h, followed by overexpression with ORF9b (SARS2) for an additional 24 h. Cells were then stimulated with 10 µm RO8191 for 12 h. The mRNA expression levels of *ISG15* (O), *ISG56* (P), and *IFITM1* (Q) were determined by RT‐qPCR. (R‐T) PPM1A‐knockout (KO) cells (sgPPM1A#1) and non‐targeting sgRNA (sgNT) control cells were overexpressed with ORF9b (SARS2) for 24 h, followed by stimulation with 10 µm RO8191 for 12 h. The mRNA expression levels of *ISG15* (R), *ISG56* (S) and *IFITM1* (T) were determined by RT‐qPCR (top), and the corresponding protein levels were assessed by immunoblotting (bottom). Data in (F‐M) and (O‐T) were presented as mean ± SEM in biological triplicates. *P* values in (F) and (G) were calculated by two‐way ANOVA with Šidák's multiple comparisons tests. *P* values in (H‐K) and (O‐T) were calculated by Student's *t*‐tests, while *P* values in (L) and (M) were calculated by one‐way ANOVA with Dunnett's multiple comparisons tests. ns, not significant. *P < 0.05, **P < 0.01, ***P < 0.001.

Given that ORF9b suppressed STAT2 phosphorylation and interacted with PPM1A, we speculated that ORF9b might hijack PPM1A to downregulate STAT2 phosphorylation. The in vitro pull‐down assay revealed that PPM1A directly interacted with STAT2 (Figure 6A,B). The IF data further confirmed their co‐localization within cells (Figure 6C). Furthermore, PPM1A exhibited high binding affinity to STAT2, with a binding constant of 70.9 nm as demonstrated by SPR assays (Figure 6D). Specifically, the overexpression of PPM1A significantly reduced STAT2 phosphorylation upon stimulation with RO8191 (Figure 6E and Figure S7A). Densitometric quantification from three independent biological replicates confirmed that PPM1A overexpression significantly decreased the p‐STAT2/total‐STAT2 ratio at 30 min (min), 1 h, and 2 h post‐RO8191 stimulation, whereas the p‐STAT1/total‐STAT1 ratio was not significantly altered (Figure 6F,G and Figure S7B–E). Because RO8191 directly activates IFNAR2 and recombinant IFN‐β directly binds IFNAR1/IFNAR2, both stimuli trigger JAK‐STAT signaling at the receptor level without requiring endogenous IFN production, thereby isolating the IFN‐mediated signaling layer from the IFN induction layer. Consistently, PPM1A overexpression suppressed ISG15 and ISG56 expression at both mRNA and protein levels upon stimulation with RO8191 or IFN‐β (Figure 6H–K). The co‐overexpression of sarbecovirus ORF9b homologs with PPM1A further suppressed the expression of *ISG15* and *ISG56* compared to PPM1A overexpression only (Figure 6L,M). In vitro pull‐down assays confirmed the simultaneous association of ORF9b, PPM1A, and STAT2, supporting the formation of a ternary complex (Figure 6N). In addition, PPM1A and STAT2 simultaneously co‐localized with various ORF9b homologs, confirming their pairwise interactions (Figure S8A).

The antiviral function of STAT2 requires both phosphorylation‐mediated activation and subsequent nuclear translocation [37]. Given that PPM1A bound STAT2 with high affinity, we speculated that such tight binding might retain STAT2 in the cytoplasm, thereby preventing its nuclear import. Therefore, we co‐expressed STAT2‐GFP with PPM1A‐RFP in HEK293T cells, followed by RO8191 stimulation. In the absence of PPM1A‐RFP, STAT2‐GFP underwent robust nuclear accumulation upon RO8191 treatment (Figure S8B). In contrast, PPM1A‐RFP co‐expression markedly restricted STAT2 to the cytoplasm, indicating that PPM1A physically sequestered STAT2 and blocked its nuclear translocation. Our above results demonstrated that PPM1A overexpression suppressed RO8191‐induced ISG expression and STAT2 phosphorylation. To determine whether this immune antagonism depended on the catalytic activity of PPM1A, we generated a phosphatase‐dead mutant. Asp239 (D239) is an essential residue that coordinates both M1 and M3 metal ions in the PPM1A trinuclear metal center, and its substitution with asparagine (N) abolishes catalytic activity [34]. We constructed the PPM1A (D239N) mutant and compared it with PPM1A (WT). Unlike PPM1A (WT), PPM1A (D239N) did not suppress *ISG15* or *ISG56* mRNA expression upon RO8191 stimulation (Figure S8C,D). Consistently, PPM1A (D239N) was unable to reduce STAT2 phosphorylation at any of the time points examined (Figure S8E). To test whether PPM1A could directly dephosphorylate STAT2 Tyr690, we enriched Tyr690‐phosphorylated STAT2 from RO8191‐stimulated cells (Figure S8F). Tyr690 is the canonical JAK‐mediated phosphorylation site essential for STAT2 activation [37]. In an in vitro phosphatase assay, incubation of phosphorylated STAT2 with purified recombinant PPM1A did not reduce Tyr690 phosphorylation, consistent with PPM1A belonging to the PP2C family of metal‐dependent Ser/Thr phosphatases (Figure S8G). Given that PPM1A (D239N) failed to reduce STAT2 phosphorylation in cells, we speculated that PPM1A likely modulated STAT2 Tyr690 phosphorylation indirectly through Ser/Thr residues on STAT2. Together with the cytoplasmic retention observed above, these data supported a dual mechanism in which PPM1A suppressed JAK‐STAT signaling through both physical sequestration of STAT2 in the cytoplasm and catalysis‐dependent, indirect downregulation of STAT2 Tyr690 phosphorylation.

To further determine whether the inhibitory effect of ORF9b on JAK‐STAT2 signaling was dependent on PPM1A, we performed the loss‐of‐function assay utilizing siRNA to knock down (KD) or sgRNA to knock out (KO) endogenous PPM1A (Figure S8H–J). Both the negative control siRNA (siNC)‐ and siPPM1A‐transfected A549 cells were overexpressed with ORF9b followed by RO8191 stimulation. The results revealed that the expression levels of *ISG15*, *ISG56*, and *IFITM1* were significantly elevated in PPM1A‐KD cells compared with those in control cells expressing ORF9b alone (Figure 6O–Q). Consistently, PPM1A KO in A549 cells also resulted in the upregulation of ISG expression at both mRNA and protein levels following RO8191 stimulation, regardless of ORF9b overexpression (Figure 6R–T). To confirm that these phenotypes were specifically attributable to the loss of PPM1A, we performed complementation by re‐expressing PPM1A in PPM1A‐KO cells. Re‐expression of PPM1A significantly restored the suppression of *ISG15*, *ISG56*, and *IFITM1* in PPM1A‐KO cells (Figure S8K–M). Altogether, these results demonstrated that ORF9b hijacked PPM1A to dampen downstream innate immune signaling through the catalysis‐dependent, indirect downregulation of STAT2 phosphorylation.

### 2.7 PPM1A Dephosphorylates ORF9b to Facilitate Its Immunosuppressive Activity

Considering that the ORF9b‐PPM1A interaction and the ORF9b phosphorylation state regulated the antagonistic capacity of ORF9b, we further characterized the functional relationship in ORF9b phosphorylation and phosphatase PPM1A. Previous research has established that ORF9b competes with HSP90 for binding to TOM70, thereby impairing the subsequent recruitment of TBK1 and IRF3 and suppressing the innate immune signaling [28, 31, 68]. Importantly, phosphorylated ORF9b loses its ability to bind TOM70, resulting in restored innate immune activation [21, 50]. To verify the role of phosphorylation on ORF9b function, we generated phosphomimetic (Ser substituted with Glu) and phospho‐deficient (Ser substituted with Ala) mutants at two well‐characterized phosphorylation sites, namely S50 and S53 [21, 26, 31]. We first assessed whether the Ser‐to‐Glu and Ser‐to‐Ala substitutions perturb the overall conformation of ORF9b. We performed AlphaFold 3 structure prediction of wild‐type ORF9b and of each mutant (Figure S9A–G). Subsequently, we calculated the change in folding stability (ΔΔG) and the deviation in backbone‐related energy terms using Rosetta. We then superimposed each mutant model onto wild‐type ORF9b and calculated the global and local (residues within 5 Å of the mutated site) Cα RMSD. The global Cα RMSD ranged from 0.381 to 0.598 Å and the local RMSD from 0.126 to 0.239 Å, with the local RMSD consistently smaller than the global RMSD. ΔΔG of the single and double mutants ranged from ‐1.374 to +1.857 REU, and the backbone‐related terms deviated by no more than 0.522 REU, indicating that none of these substitutions markedly destabilized ORF9b or perturbed its backbone geometry. Furthermore, all phosphomimetic mutants significantly upregulated the expression levels of *IFNB1*, *ISG15*, and *ISG56* compared to wild‐type ORF9b, although all variants still retained partial inhibitory capacities relative to empty vector controls (Figure S9H–J). Conversely, phospho‐deficient S53A and S50A/S53A mutants largely preserved the immunosuppressive activity, with the S50A single mutant showing a modest enhancement of *IFNB1* and *ISG56* expression (Figure S9K–M). These data collectively demonstrated that the ORF9b‐mediated antagonism of the innate immune signaling relied on its phosphorylation state.

**FIGURE 7:**
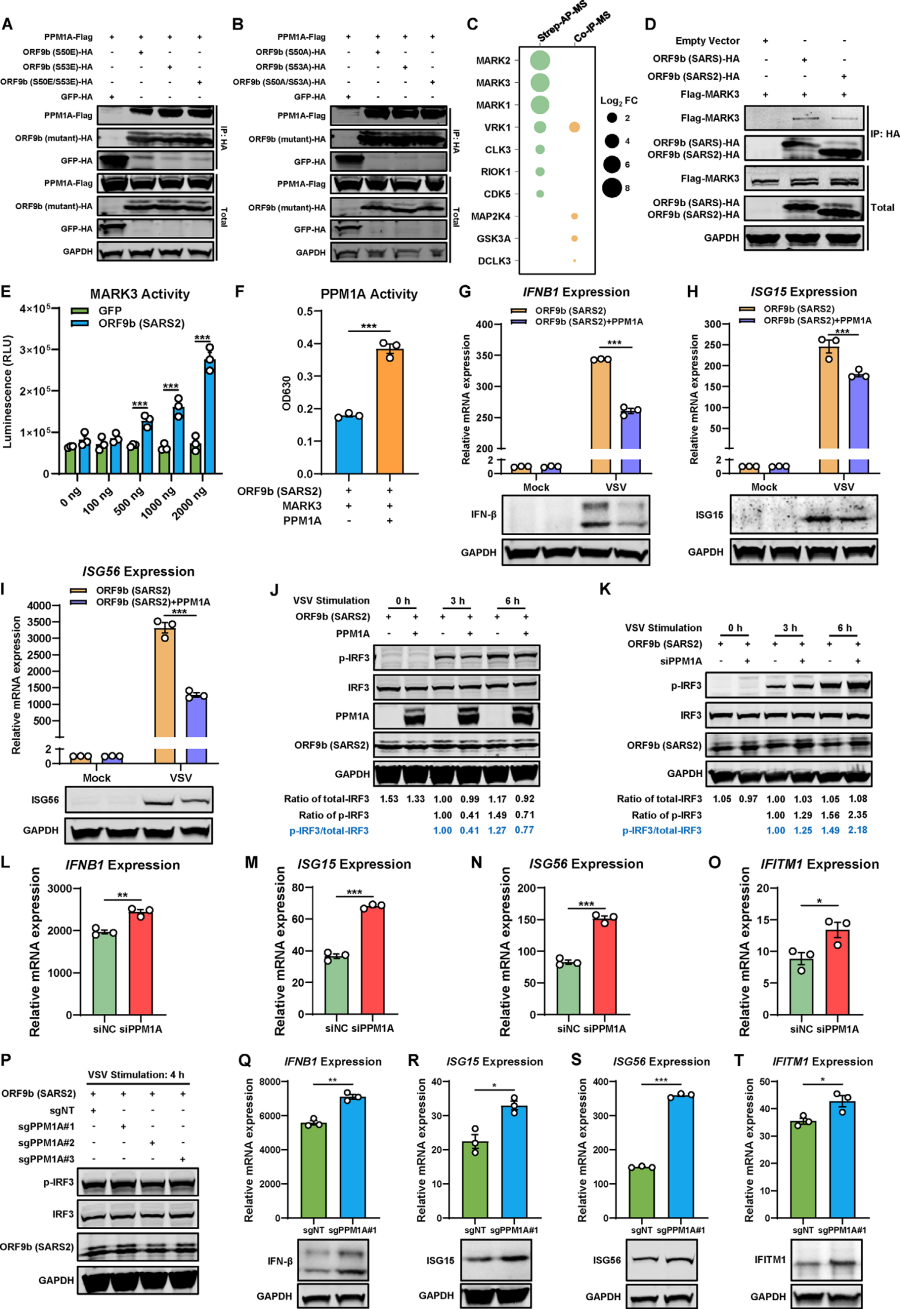
ORF9b engages PPM1A to reduce its phosphorylation state. (A, B) HA‐tagged phosphomimetic and phospho‐deficient ORF9b (SARS2) mutants were co‐overexpressed with Flag‐tagged PPM1A in HEK293T cells, respectively, followed by anti‐HA Co‐IP assays. (C) Kinases enriched by SARS‐CoV‐2 ORF9b were identified across three proteomic approaches. Only candidates with ≥ 1.5‐fold enrichment in Strep‐AP‐MS and Co‐IP‐MS assays were included in the visualization. No kinases met the previously defined ≥ 2‐fold cutoff for SPIDER‐MS, and therefore none from this dataset were displayed. (D) Co‐IP assays were performed to verify interactions between MARK3 and ORF9b (SARS) as well as ORF9b (SARS2). (E) In vitro MARK3 kinase assays were performed using ORF9b (SARS2) proteins as substrates with increasing protein amounts. GFP proteins were treated as negative control. (F) ORF9b proteins were phosphorylated via the in vitro MARK3 assay, followed by incubation with recombinant PPM1A proteins to perform in vitro phosphatase experiment. Free phosphate radical was detected by Malachite Green Phosphate Detection Kit. (G‐I) HEK293T cells were overexpressed with ORF9b from SARS‐CoV‐2 alone or in combination with PPM1A for 24 h, followed by VSV infection for 12 h. mRNA expression of *IFNB1* (G), *ISG15* (H), *ISG56* (I) were determined by RT‐qPCR (top), and the corresponding protein levels in cell lysates were assessed by immunoblotting (bottom). (J) A549 cells overexpressing ORF9b (SARS2) alone or with PPM1A were infected with VSV for indicated times, followed by western blot analysis with antibodies against total IRF3 and p‐IRF3. Densitometric quantifications of total‐IRF3, p‐IRF3, and p‐IRF3/total‐IRF3 were shown. (K) A549 cells were transfected with PPM1A‐targeting or negative control (NC) siRNAs for 12 h, followed by overexpression with ORF9b (SARS2) for 24 h. Cells were further infected with VSV for 0, 3, and 6 h prior to western blot analysis with antibodies against total IRF3 and p‐IRF3. Densitometric quantifications of total‐IRF3, p‐IRF3, and p‐IRF3/total‐IRF3 were shown. (L‐O) A549 cells were treated as in (K), except that cells were infected with VSV for 12 h. The mRNA expression levels of *IFNB1* (L), *ISG15* (M), *ISG56* (N), and *IFITM1* (O) within siNC‐ and siPPM1A‐treated cells were quantified via RT‐qPCR. (P) PPM1A‐knockout (KO) A549 cells were generated using three independent sgRNAs (sgPPM1A#1, sgPPM1A#2, and sgPPM1A#3). The negative control cells were treated with non‐targeting sgRNA (sgNT). Both PPM1A‐KO and control cells were overexpressed with ORF9b (SARS2) for 24 h, followed by infection with VSV for 4 h. The expression levels of total IRF3 and p‐IRF3 within these cells were analyzed with western blot. (Q–T) Both sgNT‐ and sgPPM1A#1‐treated A549 cells were overexpressed with ORF9b (SARS2) for 24 h, followed by infection with VSV for an additional 12 h. The mRNA expression levels of *IFNB1* (Q), *ISG15* (R), *ISG56* (S), and *IFITM1* (T) within cells were quantified via RT‐qPCR (top), and the corresponding protein levels in cell lysates were assessed by immunoblotting (bottom). Data in (E–I), (L–O) and (Q–T) were presented as mean ± SEM in biological triplicates. *P* values in (E) and (G–I) were calculated by two‐way ANOVA with Šidák's multiple comparisons tests, while *P* values in (F), (L–O) and (Q–T) were calculated by Student's *t*‐tests. *P < 0.05, **P < 0.01, ***P < 0.001.

Subsequently, we examined whether ORF9b phosphorylation influenced its interaction with PPM1A. Interestingly, Co‐IP assays revealed that PPM1A robustly interacted with all six ORF9b mutants, including both phosphomimetic and phospho‐deficient variants (Figure 7A,B). IF assays further confirmed the co‐localization of PPM1A with these mutants, indicating that the binding of PPM1A to ORF9b was independent of the ORF9b phosphorylation state (Figure S10A,B). Although ORF9b phosphorylation sites are well‐documented, the responsible kinase and phosphatase have not been identified [18, 29, 40, 41, 45, 49]. Therefore, we sought to identify host kinases responsible for ORF9b phosphorylation. Proteomic analyses using Co‐IP‐MS and Strep‐AP‐MS revealed that multiple candidate kinases potentially interacted with ORF9b (Figure 7C). Specifically, Strep‐AP‐MS identified MARK1, MARK2, MARK3, vaccinia‐related kinase 1 (VRK1), CDC‐like kinase 3 (CLK3), RIO kinase 1 (RIOK1), and cyclin‐dependent kinase 5 (CDK5), whereas Co‐IP‐MS captured VRK1, mitogen‐activated protein kinase kinase 4 (MAP2K4), glycogen synthase kinase‐3 alpha (GSK3A), and doublecortin‐like kinase 3 (DCLK3). SPIDER‐MS did not detect kinases above the predefined threshold. Based on these observations, we selected four candidate kinases (MARK1, MARK2, and MARK3 from the Strep‐AP‐MS dataset, and GSK3A from the Co‐IP‐MS dataset) and performed Co‐IP assays to examine the interaction between these kinases and ORF9b. The results showed that MARK3 robustly interacted with ORF9b proteins of both SARS‐CoV and SARS‐CoV‐2, whereas no interaction was observed for MARK1 or GSK3A (Figure 7D and Figure S10C,D). MARK2 was not successfully expressed and was therefore not shown.

To verify whether MARK3 and PPM1A regulated ORF9b phosphorylation states, we performed the in vitro kinase assay and phosphatase assay due to the absence of available antibodies against phosphorylated ORF9b. Newly generated ADP from reacted ATP in the MARK3 kinase assay reflected the phosphorylation of substrates, which was detected by subsequent luminescence assay (Figure S10E). The results demonstrated that MARK3 specifically phosphorylated ORF9b, as evidenced by substrate‐specific luminescence increases (Figure 7E). Conversely, the completely phospho‐deficient mutant ORF9b (p8M), all eight previously reported phosphorylation sites (Y42, S50, S53, S63, T72, T83, T84, and T95) of which were substituted with alanine residues, was resistant to MARK3‐mediated phosphorylation (Figure S10F). Structural prediction of ORF9b (p8M) by AlphaFold 3 and Rosetta calculation yielded a global Cα RMSD of 0.630 Å, a local RMSD of 0.368 Å, a ΔΔG of +10.828 REU, and a backbone‐related energy deviation of 0.820 REU (Figure S11A,B). The ΔΔG was larger than that of any single or double mutant, which is expected from the simultaneous loss of side chain‐mediated interactions at eight sites. The backbone‐related energy deviation remained of the same order of magnitude as those of the single and double mutants, indicating that the substitutions did not distort the ORF9b backbone. To identify the MARK3‐targeted phosphorylation sites on ORF9b, we also constructed and purified eight single‐site revertant mutants based on ORF9b (p8M) (Figure S11C–J). Interestingly, all revertant mutants were phosphorylated by MARK3 to varying extents, with p8M‐A42Y, p8M‐A50S, p8M‐A53S, p8M‐A83T, and p8M‐A84T exhibiting higher degrees of phosphorylation (Figure S11K,L). These findings indicated that MARK3 might not act on a single specific residue but rather regulated ORF9b phosphorylation through coordinated modification of multiple sites. To assess whether PPM1A directly targeted ORF9b phosphorylation, we performed in vitro phosphatase assays utilizing MARK3‐generated phosphorylated ORF9b (Figure S11M). The released phosphates from phospho‐substrates were detected with the malachite green phosphate detection reagent. The color intensity of generated blue‐green complexes was proportional to the amounts of phosphates released by PPM1A‐mediated dephosphorylation. Results revealed elevated levels of free phosphates in the presence of wild‐type PPM1A, indicating that PPM1A acted as a phosphatase directly catalyzing ORF9b dephosphorylation (Figure 7F). In contrast, the catalytically inactive D239N mutant did not significantly alter free phosphate levels, confirming that the dephosphorylation depended on the catalytic activity of PPM1A (Figure S11N). Notably, all the ORF9b potential phosphorylation sites reside in flexible N‐terminal and loop regions that are spatially distinct from the docking interface. This spatial separation supports a dual‐recognition model in which the D16‐R186, R58‐E35, and Q20‐N188/G189 contacts provide the primary binding energy and properly orient ORF9b, thereby presenting its Ser/Thr cluster to the PPM1A catalytic pocket for dephosphorylation. Taken together, our results demonstrated that MARK3 interacted with and phosphorylated ORF9b, while PPM1A directly dephosphorylated ORF9b to preserve its unphosphorylated state, thereby maintaining the capacity of ORF9b to antagonize host innate immune responses.

### 2.8 Sarbecovirus ORF9b Recruits PPM1A to Antagonize Innate Immune Responses

To determine how this phosphorylation‐dephosphorylation cycle contributes to the suppression of interferon induction through the RIG‐I/MAVS‐TBK1‐IRF3 axis, we next co‐expressed ORF9b homologs with PPM1A and monitored TBK1‐IRF3 signaling and IFN/ISG expression upon VSV infection. Co‐expression of ORF9b homologs derived from SARS‐CoV‐2 with PPM1A further suppressed the induction of IFN‐β, ISG15, and ISG56 at both mRNA and protein levels upon VSV infection compared to ORF9b expression alone (Figure 7G–I and Figure S12A). The suppressive effect was conserved across ORF9b homologs derived from SARS‐CoV and bat coronaviruses, as shown by the reduced *IFNB1*, *ISG15*, and *ISG56* mRNA levels upon PPM1A co‐expression (Figure S12B–G). To determine whether this enhancement of immune suppression required the catalytic activity of PPM1A, we compared PPM1A (WT) with the catalytically inactive PPM1A (D239N). PPM1A (D239N) did not suppress *IFNB1*, *ISG15*, or *ISG56* expression upon ORF9b co‐expression and VSV infection, indicating that the catalytic activity of PPM1A was required for its function in the TBK1‐IRF3 axis (Figure S12H–J). In line with these observations, the phosphorylation of IRF3 was consistently attenuated at both 3 and 6 h post‐infection in the PPM1A and ORF9b co‐expression group (Figure 7J and Figure S12K). Densitometric quantification revealed that PPM1A co‐expression reduced the p‐IRF3/total‐IRF3 ratio by approximately 59% at 3 h and 40% at 6 h relative to ORF9b expression alone, indicating that PPM1A‐mediated suppression of IRF3 phosphorylation was more pronounced at the early stage of infection. These results indicated that the interaction between ORF9b and PPM1A enhanced the suppression of type I interferon signaling, further dampening the host antiviral response.

To further determine whether ORF9b‐mediated immune suppression required PPM1A, we examined the effects of PPM1A depletion on IRF3 phosphorylation and downstream antiviral gene expression. siRNA‐mediated knockdown of PPM1A in A549 cells restored IRF3 phosphorylation, with the p‐IRF3/total‐IRF3 ratio increased by approximately 25% at 3 h and 46% at 6 h compared to negative controls, suggesting that the cumulative impact of PPM1A suppression became increasingly evident over the course of infection (Figure 7K). PPM1A knockdown also upregulated the expression of *IFNB1*, *ISG15*, *ISG56*, and *IFITM1* after ORF9b overexpression and subsequent VSV infection, indicating that the antagonistic effect of ORF9b on innate immune responses was diminished in the absence of PPM1A (Figure 7L–O). To further validate these observations, we generated three PPM1A‐KO A549 cell lines using independent single‐guide RNAs (sgPPM1A). PPM1A KO increased IRF3 phosphorylation upon ORF9b overexpression and VSV infection (Figure 7P). The expression levels of IFN‐β, ISG15, ISG56, and IFITM1 at both the mRNA and protein levels were also significantly elevated in PPM1A‐KO cells compared to non‐targeting sgRNA (sgNT)‐transduced cells (Figure 7Q–T and Figure S12L). Complementation by re‐expressing PPM1A in PPM1A‐KO cells reversed this phenotype, restoring the suppression of *IFNB1*, *ISG15*, *ISG56*, and *IFITM1* mRNA expression (Figure S12M–P). These findings suggested that PPM1A was a critical host factor hijacked by sarbecovirus ORF9b to suppress innate immune signaling. The universal recruitment of PPM1A across sarbecovirus ORF9b homologs underscored an evolutionarily conserved strategy to antagonize antiviral defenses.

### 2.9 PPM1A Inhibitor Restores Antiviral Innate Immune Homeostasis

**FIGURE 8:**
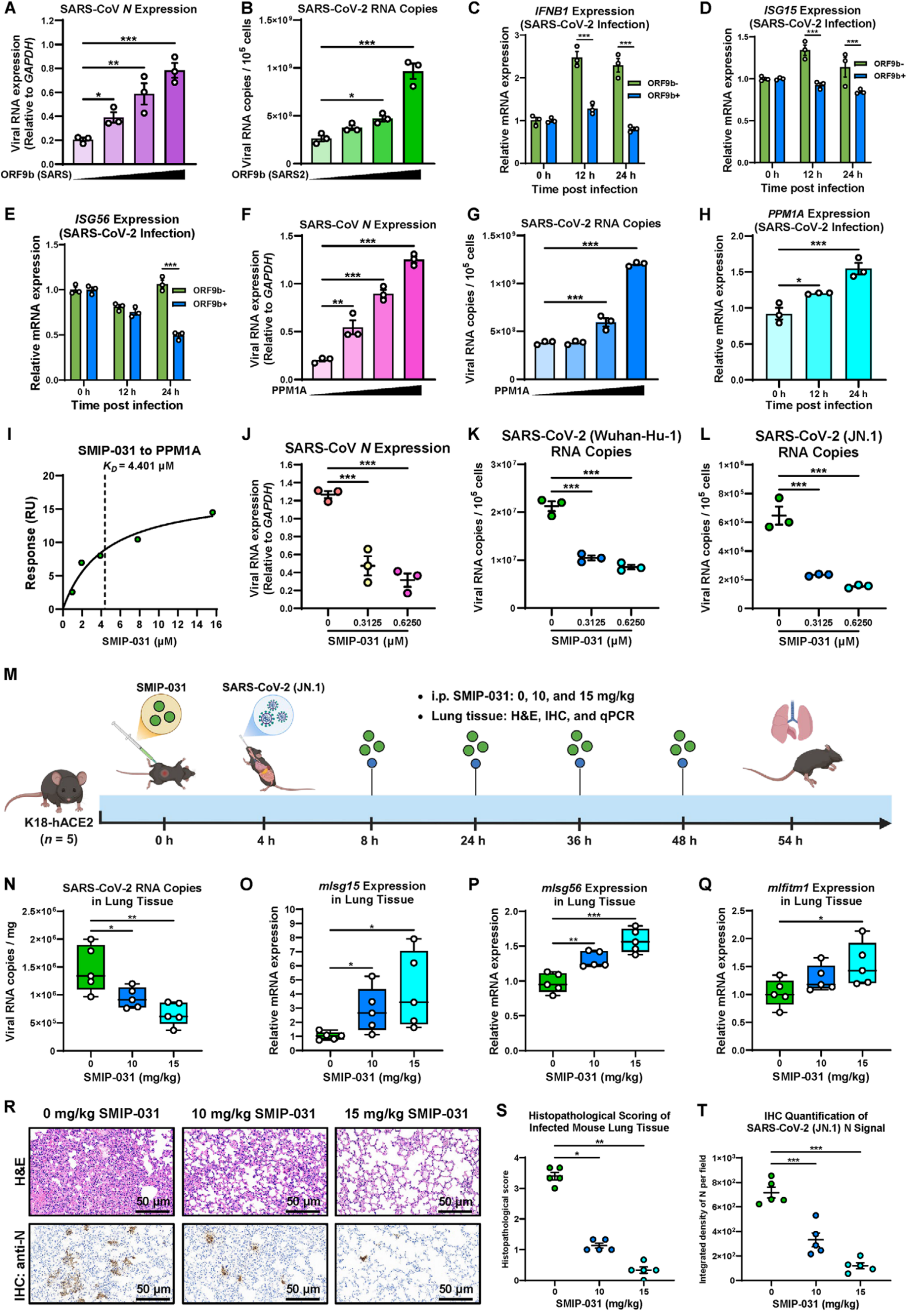
PPM1A inhibitor promotes the re‐establishment of innate immune homeostasis. (A, B) HEK293T‐hACE2 cells were overexpressed with increasing amounts of ORF9b (SARS) and ORF9b (SARS2), respectively, followed by infection with corresponding authentic viruses. The mRNA expression levels of intracellular SARS‐CoV *N* were measured using RT‐qPCR and normalized relative to those of *GAPDH* (A). The intracellular SARS‐CoV‐2 RNA copies were quantified via one‐step SARS‐CoV‐2 RNA detection kits and normalized relative to 100,000 cells (B). (C‐E) HEK293T‐hACE2 cells were overexpressed with ORF9b (SARS2) for 24 h, followed by infection with SARS‐CoV‐2. The relative mRNA expression levels of *IFNB1* (C), *ISG15* (D), and *ISG56* (E) were quantified at 0, 12, and 24 h post‐infection. (F, G) HEK293T‐hACE2 cells were overexpressed with increasing amounts of PPM1A, followed by infection with authentic SARS‐CoV (F) or SARS‐CoV‐2 (G). The intracellular viral RNA expression levels were quantified as in (A‐B). (H) RT‐qPCR analysis of relative *PPM1A* mRNA expression at 0, 12, and 24 h post‐SARS‐CoV‐2 infection. (I) SPR analysis of the binding affinity between recombinant PPM1A proteins and SMIP‐031 molecules. PPM1A proteins were immobilized on the CM7 sensor chip, while SMIP‐031 was used as the analyte. (J) Huh7 cells were co‐cultured with indicated concentrations of SMIP‐031 and authentic SARS‐CoV for 24 h. The relative expression levels of SARS‐CoV *N* were determined as in (A). (K, L) HEK293T‐hACE2 cells were co‐cultured with indicated concentrations of SMIP‐031 and SARS‐CoV‐2 Wuhan‐Hu‐1 (K) or JN.1 (L) for 24 h. Viral RNA copies were quantified as in (B). (M) Schematic of the in vivo animal experiment utilizing K18‐hACE2 transgenic mice. K18‐hACE2 mice (*n* = 5 per group) were intraperitoneally (i.p.) administered with 0, 10, or 15 mg/kg of SMIP‐031 at 4 h prior to infection. Each mouse was intranasally (i.n.) challenged with 1×10^5^ focus‐forming units (FFUs) of authentic SARS‐CoV‐2 (JN.1), followed by additional treatments with SMIP‐031 at 8, 24, 36, 48 h after the primary treatment. At 50 h post‐infection, all mice were euthanized and proceeded to viral RNA quantification and histopathological analysis of lung tissues. (N) Viral RNA copies per gram of lung tissues from SMIP‐031‐treated mice were quantified via one‐step SARS‐CoV‐2 RNA detection kits. (O–Q) RT‐qPCR analysis of mRNA expression levels of *mIsg15* (O), *mIsg56* (P) and *mIfitm1* (Q) in lung tissues from different groups. (R) Histopathological analysis of mouse lung tissues by hematoxylin and eosin (H&E) staining and immunohistochemistry (IHC) with SARS‐CoV‐2 N antibodies. Scale bar, 50 µm. (S) Histopathological scoring of lung tissue sections from infected K18‐hACE2 mice. Semi‐quantitative grading was performed by three independent observers blinded to treatment allocation: 0, within normal limits; 1, minimal and localized alteration; 2, mild but clearly identifiable lesions; 3, moderate and multifocal pathology; 4, severe and diffusely distributed damage; and the scores were averaged to yield a single value per animal. (T) IHC quantification of SARS‐CoV‐2 (JN.1) N protein signal in lung tissues. Integrated density of N staining per field was quantified using ImageJ. Data in (A–H) and (J–L) were presented as mean ± SEM in biological triplicates, while data in (N‐Q), (S) and (T) were presented as mean ± SEM in biological quintuplicates. *P* values in (A,B), (F–H), (J–L), (N–Q), and (T) were calculated by one‐way ANOVA with Dunnett's multiple comparisons tests. *P* values in (C–E) were calculated by two‐way ANOVA with Šidák's multiple comparisons tests, while the *P* values in (S) were calculated by Kruskal‐Wallis test followed by Dunn's multiple comparisons tests. *P < 0.05, **P < 0.01, ***P < 0.001.

Our above results collectively demonstrated that sarbecovirus ORF9b acted as a conserved antagonist of innate immunity. The cellular phosphatase PPM1A was hijacked by ORF9b to orchestrate a dual‐layered suppression of innate immune responses, not only by directly dephosphorylating ORF9b to safeguard its dephosphorylated state but also by the catalysis‐dependent, indirect downregulation of STAT2 phosphorylation. To further clarify their impacts on coronavirus infection and assess the antiviral potential of targeting this axis, we employed both authentic virus infection models and pathway‐specific agonists to interrogate upstream and downstream events within the innate immune signaling cascade. ORF9b from both SARS‐CoV and SARS‐CoV‐2 markedly promoted viral replication of corresponding authentic viruses in dose‐dependent manners (Figure 8A,B). Consistent with our previous observations, the expression levels of *IFNB1*, *ISG15*, and *ISG56* were significantly suppressed in the presence of SARS‐CoV‐2 ORF9b at both 12 and 24 h post‐authentic virus infection (Figure 8C–E). Previous studies have shown that ORF9b is incorporated into SARS‐CoV virions [23]. We therefore examined whether ORF9b was also present in SARS‐CoV‐2 viral particles. Purified virions from supernatants of infected Vero E6 cells displayed intact crown‐like morphologies under negative‐staining transmission electron microscopy (TEM) (Figure S13A). Further immunogold electron microscopy (iEM) analyses utilizing SARS‐CoV‐2 N‐ and ORF9b‐specific antibodies revealed that both N and ORF9b proteins were specifically incorporated within these viral particles (Figure S13B,C). The western blot analysis with antibodies against ORF9b further confirmed the presence of ORF9b within purified SARS‐CoV‐2 virions (Figure S13D). Moreover, Co‐IP assays revealed that ORF9b specifically interacted with the N structural protein, but not with S, E, or M proteins, suggesting that ORF9b might be selectively packaged into coronavirus particles through its association with N proteins (Figure S13E). Taken together, these findings indicated that ORF9b could be a component of infectious coronavirus particles, potentially enabling its immune‐antagonistic function at the early stage of infection.

Our previous analyses utilizing clinical RNA‐seq datasets have shown that the expression levels of *PPM1A* were positively associated with the severity of COVID‐19 and negatively correlated with the expression of *ISG15*. We therefore clarified the relationship between PPM1A expression and coronavirus infection. Results revealed that the overexpression of PPM1A promoted the replication of both SARS‐CoV and SARS‐CoV‐2 in dose‐dependent manners (Figure 8F,G). Furthermore, SARS‐CoV‐2 infection also resulted in elevated expression of *PPM1A* (Figure 8H). Conversely, the overexpression of MARK3 kinases dose‐dependently inhibited SARS‐CoV and SARS‐CoV‐2 replication (Figure S13F,G). Given the lack of specific pharmacological inhibitors directly targeting ORF9b and the essential role of PPM1A as a critical cofactor for ORF9b function, we speculated that targeting PPM1A could represent a potential antiviral strategy against coronavirus infection. We have previously developed a small‐molecule PPM1A inhibitor SMIP‐031, which potently restricts *Mycobacterium tuberculosis* infection [69]. The SPR assay confirmed that SMIP‐031 directly bound to PPM1A with a binding constant of 4.401 µm (Figure 8I). To evaluate the antiviral potential of SMIP‐031, we co‐treated susceptible cells with both SMIP‐031 and authentic coronaviruses. The results showed that treatment with SMIP‐031 at 0.3125 or 0.625 µm significantly suppressed viral replication in SARS‐CoV‐infected Huh7 cells and SARS‐CoV‐2 (Wuhan‐Hu‐1 and JN.1)‐infected HEK293T‐hACE2 cells (Figure 8J–L). To assess whether SMIP‐031 acted through PPM1A rather than off‐target molecules, we compared its antiviral activity in PPM1A‐knockout (sgPPM1A) and non‐targeting control (sgNT) HEK293T cells overexpressing human ACE2 and infected with SARS‐CoV‐2 (JN.1). SMIP‐031 significantly reduced viral RNA copies in sgNT cells, whereas PPM1A knockout alone also reduced viral RNA relative to untreated sgNT cells, consistent with the essential role of PPM1A as a cofactor for ORF9b function (Figure S13H). Importantly, SMIP‐031 produced no additional reduction in sgPPM1A cells at either concentration. The viral RNA levels in SMIP‐031‐treated sgNT cells were not significantly different from those in untreated sgPPM1A cells, indicating that its antiviral activity required PPM1A (Figure S13H). Conversely, the treatment with PCC0208017, the pharmacological inhibitor of MARK3, resulted in increased viral replication of both SARS‐CoV and SARS‐CoV‐2 (Figure S13I,J). These findings suggested that targeting PPM1A could be exploited to restore antiviral homeostasis.

To evaluate the protective efficacy of the PPM1A inhibitor SMIP‐031 in a prophylactic setting, K18‐hACE2 transgenic mice (*n* = 5 per group) were intraperitoneally (i.p.) administered with 10 or 15 mg kg^−1^ of SMIP‐031 prior to SARS‐CoV‐2 (JN.1) infection (Figure 8M). At 4 h post‐treatment, each mouse was intranasally (i.n.) challenged with 1×10^5^ focus‐forming units (FFUs) of authentic viruses, followed by continuous treatments with SMIP‐031. At 50 h post‐infection, all mice were euthanized for lung tissue analysis. Results demonstrated that both 10and 15 mg kg^−1^ doses of SMIP‐031 significantly inhibited SARS‐CoV‐2 replication in lung tissues compared to mice treated with vehicle (Figure 8N). Additionally, the expression levels of mouse *Isg15* (*mIsg15*), *mIsg56*, and *mIfitm1* were significantly elevated following SMIP‐031 administration, suggesting restored antiviral innate immune responses in vivo (Figure 8O–Q). Further histopathological examinations showed that SARS‐CoV‐2‐infected mice exhibited severe pulmonary pathology, characterized by collapsed alveoli, thickened alveolar septa, and extensive infiltration of inflammatory cells (Figure 8R). Nevertheless, treatment with SMIP‐031 markedly alleviated lung injury. Immunohistochemistry (IHC) assays using antibodies against SARS‐CoV‐2 N proteins revealed substantially fewer N‐positive cells in SMIP‐031‐treated mice compared to control mice (Figure 8R). To provide quantitative support for these histopathological observations, lung tissue sections were evaluated by three independent investigators in a blinded manner using a semi‐quantitative scoring system. Histopathological scores were markedly reduced in SMIP‐031‐treated groups compared to vehicle‐treated controls (Figure 8S and Figure S13K,L). IHC signal quantification confirmed that the integrated density of SARS‐CoV‐2 N protein per field was significantly decreased in mice receiving 10 or 15 mg kg^−1^ of SMIP‐031 relative to the control group (Figure 8T). Collectively, these results demonstrated that the pharmacological inhibition of PPM1A by SMIP‐031 not only suppressed coronavirus replication but also restored the innate immune homeostasis in vivo, supporting PPM1A as a potential host‐directed antiviral target in a prophylactic setting.

## 3 Discussion

**FIGURE 9:**
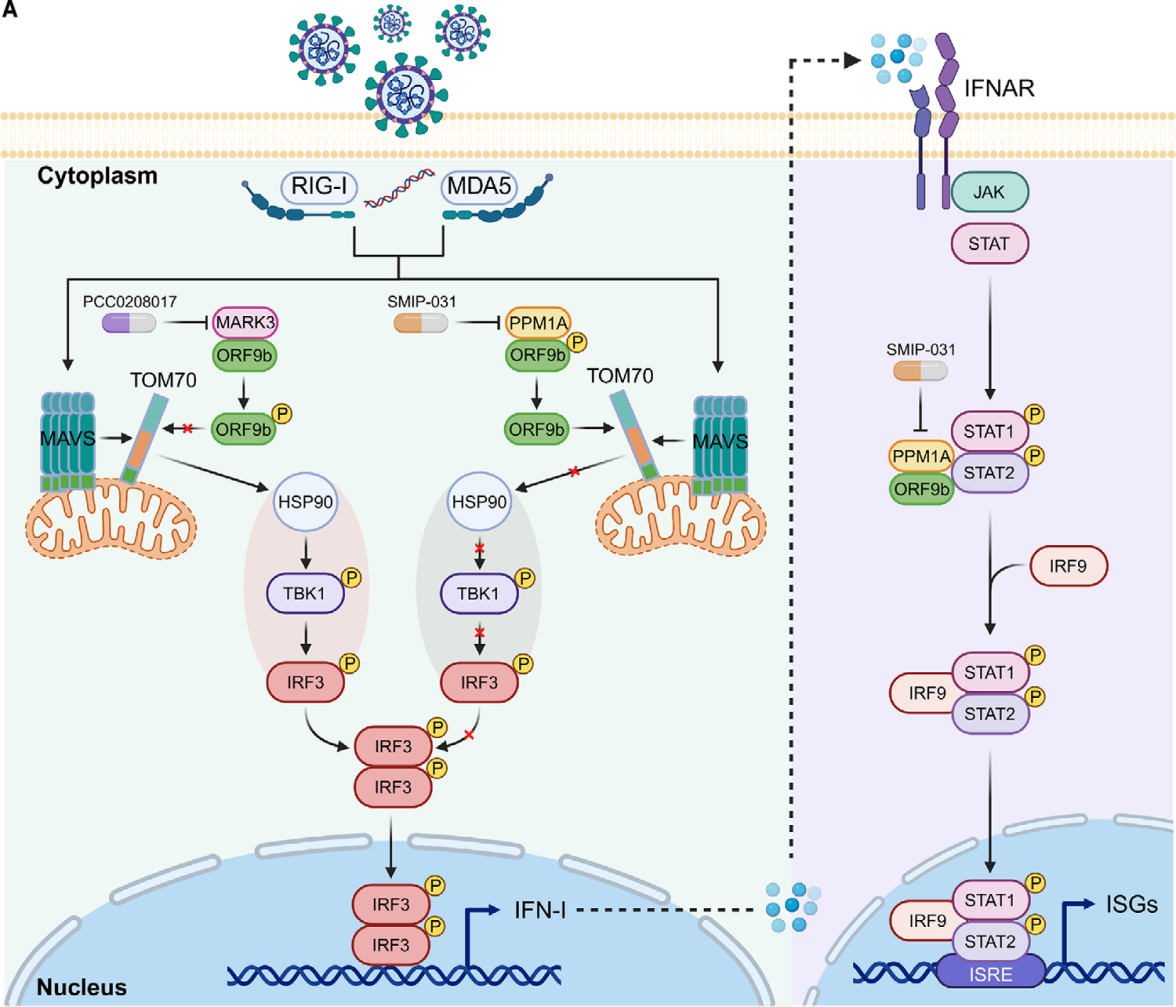
Model of ORF9b‐PPM1A‐STAT2 axis in sarbecovirus immune evasion. (A) Schematic of sarbecovirus ORF9b‐mediated innate immune evasion via recruitment of PPM1A to modulate the phosphorylation homeostasis of ORF9b and STAT2.

Innate immune antagonism is a hallmark of coronaviruses. Although the ORF9b proteins of SARS‐CoV and SARS‐CoV‐2 have previously been linked to immune evasion through distinct mechanisms, our study established a unifying perspective by identifying the ORF9b‐PPM1A axis as a conserved mechanism within the *Sarbecovirus* subgenus. Based on our investigations, we proposed a dual‐mechanism model of sarbecovirus ORF9b‐mediated innate immune evasion (Figure 9A). At the interferon induction layer, ORF9b recruits PPM1A to directly dephosphorylate itself, maintaining its TOM70‐binding activity and suppressing TBK1‐IRF3‐mediated IFN production. At the interferon signaling layer, ORF9b hijacks PPM1A to indirectly downregulate STAT2 phosphorylation, impairing JAK‐STAT‐mediated ISG expression. These two mechanisms are mechanistically independent. First, upon sarbecovirus infection, virion‐associated ORF9b proteins were released to antagonize innate immune responses at the early stage of infection. Mechanistically, MARK3 acted as an upstream kinase that phosphorylated ORF9b to inhibit its interaction with TOM70. However, ORF9b recruited the phosphatase PPM1A to directly dephosphorylate itself, which enhanced its binding to TOM70 and disrupted the TOM70‐HSP90 association, thereby resulting in reduced phosphorylation of TBK1 and IRF3 and impaired IFN‐I production. Second, ORF9b further suppressed antiviral signaling downstream of interferon regardless of its phosphorylation state. ORF9b interacted with STAT2 and hijacked PPM1A to indirectly downregulate STAT2 phosphorylation, thereby inhibiting its nuclear translocation and attenuating the expression of various ISGs. Together, these two coordinated mechanisms established the ORF9b‐PPM1A axis as a conserved strategy employed by sarbecoviruses to antagonize innate immunity at both the interferon production and interferon signaling levels. Importantly, pharmacological inhibition of PPM1A phosphatase activity by SMIP‐031 disrupted this regulatory axis and reactivated antiviral signaling. Thus, targeting PPM1A could re‐establish innate immune homeostasis during sarbecovirus infection.

Through evolutionary and conservation analyses, we revealed that ORF9b proteins were highly conserved among sarbecoviruses, including human‐, bat‐, civet‐, and pangolin‐derived strains. Bats, civets, and pangolins are recognized as potential natural or intermediate reservoirs of human coronaviruses, and both SARS‐CoV and SARS‐CoV‐2 are considered to originate from zoonotic spillover events. Therefore, investigating the conservation and function of ORF9b across animal and human sarbecoviruses is essential for understanding cross‐species transmission and assessing future pandemic risks. Previous studies have implicated ORF9b in innate immune suppression; our work advanced this understanding by identifying a universally conserved antagonistic mechanism. All verified ORF9b homologs exerted antagonistic effects on innate immune responses, targeting both upstream and downstream pathways. Both human‐ and animal‐derived coronavirus ORF9b proteins demonstrated high binding affinities to PPM1A and shared conserved binding motifs within the ORF9b‐PPM1A interaction interface. These functional and structural conservations possibly accounted for the universal mechanisms of coronavirus‐mediated immune evasion. The abundance of ORF9b has been reported to accumulate rapidly following SARS‐CoV‐2 infection [21]. In addition, SARS‐CoV‐2 Omicron sublineages present higher levels of ORF9b subgenomic RNA compared with early isolates [70]. Consistently, our study demonstrated that Omicron‐derived ORF9b variants retained inhibitory capacities on immune responses comparable to those of wild‐type ORF9b. Whether these variations in ORF9b contribute to enhanced ORF9b expression and binding affinity to PPM1A needs to be further investigated.

Our proteomic analyses revealed that ORF9b extensively impacted coronavirus infection and host antiviral responses. Notably, ORF9b selectively engaged TOM70, a mitochondrial adaptor that bridges antiviral signaling and organelle homeostasis. This finding was consistent with previous reports that ORF9b competes with HSP90 for binding to TOM70, thereby inhibiting the recruitment of the HSP90‐TBK1‐IRF3 complex to mitochondria [28, 50, 68]. Specifically, ORF9b systematically coordinated the phosphorylation‐dependent antiviral signaling. Multiple kinases, including MARK1, MARK2, MARK3, and GSK3A, as well as several phosphatases, including PPM1A, PPM1B, and the protein phosphatase 2A (PP2A) catalytic subunit PPP2CA, were also found to potentially interact with ORF9b. Further biochemical analysis confirmed the direct binding of PPM1A to ORF9b and demonstrated that MARK3 phosphorylated ORF9b in vitro. Recently, MARK2 has been found to enhance the antiviral innate immunity by phosphorylating GEF‐H1, thereby facilitating TBK1 activation [71]. Whether ORF9b modulates additional phosphorylation molecules, such as MARK2, warrants further investigation. Besides the phosphorylation regulation, the function of ORF9b has been found to be dynamically regulated by both E3 ubiquitin ligase CRL5 and deubiquitinase USP29 [51, 52]. Our proteomic analyses revealed that ORF9b potentially interacted with additional ubiquitin‐related enzymes, including UBR2, USP5 and USP22, suggesting diversified ubiquitin‐mediated regulation of ORF9b. Interestingly, our proteomic quantifications also indicated potential interactions with cGAS and STAT2. Because cGAS is a pivotal mediator of the cGAS‐STING cytosolic DNA sensing pathway, ORF9b may also impact the innate immunity against DNA viruses. Taken together, the global interactome positioned ORF9b as a conserved immune‐evasion hub that integrated metabolic regulation, phosphorylation remodeling, ubiquitin dynamics, and multi‐innate immune pathways to ensure robust antiviral suppression.

Given that ORF9b comprehensively impacts host defense‐response pathways, we focused on the phosphorylation‐centered dual regulatory strategy employed by ORF9b in this study. We demonstrated that ORF9b inhibited innate immune responses by hijacking both upstream and downstream of innate immunity. ORF9b was found to be presented within mature SARS‐CoV‐2 virions by interacting with N proteins, consistent with previous reports that ORF9b is also detected within SARS‐CoV virions [22, 23]. Therefore, ORF9b was immediately delivered into newly infected cells, facilitating early immune antagonism upon entry. Previous studies indicate that phosphorylation modulates the antagonistic function of SARS‐CoV‐2 ORF9b, as phosphomimetic mutations disrupt its interaction with TOM70 [21]. We demonstrated that ORF9b directly bound to the host phosphatase PPM1A and maintained the dephosphorylated state of ORF9b. The dephosphorylation reinforced the ORF9b‐TOM70 interaction, disrupted the TOM70‐HSP90 complex, and attenuated TBK1‐IRF3 activation, thereby truncating interferon production at the upstream signaling level [28, 50]. This function of PPM1A is consistent with its established role as a negative regulator of antiviral signaling. PPM1A dephosphorylates TBK1 at Ser172 to attenuate STING‐TBK1‐IRF3 activation [33]. Its dephosphorylation of ORF9b preserves the viral protein in an active conformation that further suppresses this same pathway. Thus, PPM1A operates at multiple levels by turning off host antiviral kinases and simultaneously maintaining viral antagonist activity to achieve convergent immune suppression. Importantly, we also uncovered an additional downstream mechanism of innate immune modulation in which ORF9b interacted with STAT2 and recruited PPM1A to indirectly downregulate STAT2 phosphorylation, impairing STAT2 nuclear translocation and ISG induction. Strikingly, ORF9b interacted with PPM1A regardless of its own phosphorylation state. This phenomenon implies that even if the phosphorylation of ORF9b partially compromises its upstream inhibitory capacity, the downstream STAT2‐targeting mechanism remains intact, ensuring robust immune suppression across multiple signaling nodes. Although MARK3 can intrinsically phosphorylate ORF9b, the precise competitive dynamics between MARK3‐mediated ORF9b phosphorylation and PPM1A‐mediated ORF9b dephosphorylation remain to be fully resolved. The two enzymes act on the same cluster of phosphorylation residues with opposing consequences for ORF9b function, so that their relative activities determine the phosphorylation equilibrium of ORF9b. This equilibrium in turn governs the capacity of ORF9b to bind TOM70 and antagonize innate immunity. Our functional data suggested that PPM1A exerted a dominant influence in the context of infection, as the expression levels of *PPM1A* were significantly upregulated upon SARS‐CoV‐2 infection and in COVID‐19 patients. This increase in PPM1A would progressively shift the equilibrium toward the dephosphorylated form of ORF9b. In contrast, whether MARK3 expression or kinase activity is temporally regulated during infection, and how the balance between the two enzymes evolves over the course of infection, were not determined in this study. Because antibodies recognizing phosphorylated ORF9b are not available, the phosphorylation state of endogenous ORF9b could not be monitored directly in infected cells. Quantitative and time‐resolved measurement of both enzyme activities, together with the development of phospho‐ORF9b‐specific reagents, will be required to define this balance. Specifically, ORF9b consistently suppressed innate immunity across various experimental conditions. These findings provide additional mechanistic insight into ORF9b phosphorylation and its contribution to innate immune antagonism.

In addition to PPM1A, several phosphatases, including PPM1B, PPM1G, protein phosphatase 4 catalytic subunit (PP4C), and PP2A, are known to negatively regulate innate immunity by directly dephosphorylating critical signaling molecules [72, 73, 74, 75]. In the present study, we focused on PPM1A on the basis of positive correlation with COVID‐19 disease severity and direct biochemical interaction with ORF9b. However, this prioritization strategy does not exclude the possibility that other phosphatases identified in our interactome screens also contribute to innate immune regulation or viral pathogenesis. Phosphatases that were negatively correlated with disease severity in our analysis, such as PPM1G and PPP1CA, may nevertheless modulate antiviral signaling through PPM1A‐independent mechanisms or under distinct infection conditions not captured in the present study. Furthermore, PPP3CA, which was selectively elevated in severe cases, may play a context‐dependent role in late‐stage immune dysregulation. Additionally, PPM1B and PPP2CA, which met the disease correlation criterion but did not exhibit detectable binding to ORF9b in our co‐immunoprecipitation assays, may still influence coronavirus infection through alternative viral or host targets. A comprehensive understanding of the broader phosphatase network engaged during coronavirus infection, including the systematic functional characterization of each candidate, remains an important direction for future investigation.

Previous studies have reported that PPM1A dephosphorylates MAVS and TBK1, thereby dampening antiviral response [76]. We proposed a conceptual framework of “phosphatase‐centric immune antagonism” in which ORF9b exploited host phosphatase activity to modulate both host signaling cascades and viral proteins. Notably, because PPM1A directly bound to STAT2, this mechanism may also be employed by other viruses, suggesting broader relevance beyond coronaviruses. Given the consistent role of sarbecovirus ORF9b in antagonizing innate immunity, direct targeting of ORF9b offers a rational strategy for the prevention and treatment of SARS‐CoV‐2 infection. Nevertheless, the current absence of ORF9b‐specific inhibitors limits the feasibility of this strategy. In this study, we identified two host proteins, MARK3 and PPM1A, that reciprocally regulated ORF9b activity. MARK3 overexpression was found to suppress SARS‐CoV and SARS‐CoV‐2 replication, suggesting that MARK3 may act as an intrinsic antiviral factor during sarbecovirus infection. Moreover, therapeutic strategies aimed at enhancing MARK3 activity may offer a potential avenue for intervention, such as small‐molecule modulators or vaccine adjuvants. In contrast, PPM1A overexpression promoted sarbecovirus replication. Given the conserved interaction between ORF9b and PPM1A, targeting PPM1A may overcome the challenge of viral mutation and enable the development of broad‐spectrum antiviral therapies for combating both current and future coronavirus outbreaks. We have previously developed a PPM1A inhibitor SMIP‐031, which restricts *Mycobacterium tuberculosis* burden in mice [69, 77]. In this study, SMIP‐031 also significantly inhibited SARS‐CoV and SARS‐CoV‐2 infection in vitro. It reduced SARS‐CoV‐2 replication in mice when administered prophylactically, supporting PPM1A as a potential host‐directed antiviral target. However, these findings also highlight a central challenge for this strategy. Because PPM1A acts on a broad range of cellular substrates, its pharmacological inhibition is unlikely to be restricted to antiviral signaling. The consequences of sustained systemic inhibition in non‐immune tissues have not been examined. The therapeutic window of PPM1A inhibition therefore requires careful definition. Strategies that limit systemic exposure, such as short treatment courses, dose optimization, or tissue‐targeted delivery, may be required before this approach can be considered for clinical development.

Despite the mechanistic insights provided in this study, several limitations should be acknowledged. First, the structural analysis in this study relies on computational modeling rather than on experimental structures. Although AlphaFold 3‐based modeling and alanine‐scanning mutagenesis identified key interface residues of the ORF9b‐PPM1A complex, high‐resolution structural determination is required to fully define the molecular contacts and conformational dynamics of the complex. For the same reason, we cannot formally exclude that the Ser‐to‐Ala/Glu substitutions used to generate the phosphomimetic and phosphodeficient ORF9b mutants introduce local conformational changes independent of the phosphorylation state. AlphaFold 3 predictions indicated that these substitutions did not appreciably alter the overall conformation of ORF9b, and the mutants retained PPM1A binding while single‐site revertants restored MARK3‐mediated phosphorylation. Nevertheless, because these inferences rest on computational models and indirect biochemical readouts rather than on experimental structures, definitive exclusion of conformational artefacts will require high‐resolution structural analysis of the mutant proteins. Second, the majority of the functional experiments in this study were performed under overexpression conditions. While this approach is widely employed to dissect protein function, the expression levels of ORF9b and PPM1A in these systems may exceed those encountered during natural infection, and the stoichiometric relationships among ORF9b, PPM1A, and STAT2 may differ in a physiological context. Although the ORF9b‐PPM1A‐STAT2 ternary complex was reconstituted in vitro using purified recombinant proteins, and the direct binary interactions were measured by SPR using purified proteins, the formation of this complex at endogenous protein levels during authentic coronavirus infection was not directly demonstrated. Proximity ligation assays (PLAs) or comparable approaches performed in virus‐infected cells under biosafety level 3 (BSL‐3) containment will be required to establish this, which remains an important objective for future investigation. Third, the competitive dynamics between ORF9b, MARK3, and PPM1A, which govern the phosphorylation‐dephosphorylation equilibrium of ORF9b, remain incompletely characterized. Our data indicate that PPM1A‐mediated dephosphorylation dominates under the experimental conditions tested, but the precise temporal and quantitative interplay between MARK3‐directed phosphorylation and PPM1A‐directed dephosphorylation in infected cells has yet to be elucidated. Fourth, the mechanism by which PPM1A regulates STAT2 remains incompletely resolved. Our in vitro data indicate that PPM1A does not directly dephosphorylate STAT2 Tyr690, consistent with its Ser/Thr phosphatase specificity. Whether PPM1A regulates STAT2 through dephosphorylation of its Ser/Thr residues, through cytoplasmic sequestration via high‐affinity binding, or through a combination of both has not been experimentally distinguished. Site‐specific phospho‐STAT2 antibodies and nucleocytoplasmic shuttling assays will be required to resolve this question. Fifth, the specificity profile of SMIP‐031 has not been comprehensively assessed across the PPM/PP2C family. Although SMIP‐031 was previously identified as a PPM1A inhibitor, its activity against other members of the PPM family or related phosphatases has not been systematically profiled. However, SMIP‐031 did not further reduce coronavirus replication in PPM1A‐knockout cells, and the viral RNA levels in SMIP‐031‐treated wild‐type cells were comparable to those in untreated PPM1A‐knockout cells, indicating that its antiviral activity requires PPM1A and is unlikely to be primarily attributable to off‐target effects. A systematic selectivity profile against other PPM family members and related phosphatases is still required. Sixth, PPM1A inhibition as a host‐directed antiviral strategy requires extensive safety evaluation before clinical consideration. PPM1A is ubiquitously expressed and regulates diverse physiological processes, including cell cycle progression, stress response, and metabolism. The potential adverse consequences of systemic PPM1A inhibition, particularly in the context of acute viral infection, remain unknown and must be rigorously assessed in preclinical models. Importantly, our finding that SMIP‐031 acts through PPM1A addresses the selectivity of the compound but not the safety of inhibiting PPM1A systemically. Seventh, although we have validated the key IFN and ISG readouts at the protein level, the majority of our innate immune measurements were performed at the mRNA level. Protein‐level analysis was performed for representative ISGs and for IFN‐β in the key experiments, but not for every condition examined in this study. More comprehensive protein‐level characterization would further strengthen the quantitative assessment of downstream immune suppression. Eighth, the effect of PPM1A on STAT2 phosphorylation was established using type I IFN stimulation, including the IFNAR2 agonist RO8191 and recombinant IFN‐β. Although type I and type III IFNs converge on the shared STAT1‐STAT2‐ISGF3 signaling module, whether PPM1A similarly regulates type III IFN signaling was not directly examined in the present study and remains to be determined. Addressing these limitations will be essential for a comprehensive understanding of the ORF9b‐PPM1A‐STAT2 regulatory axis and for evaluating the therapeutic feasibility of targeting this pathway.

In summary, our study establishes the ORF9b‐PPM1A axis as a paradigm of phosphatase‐centric immune antagonism. By revealing how a conserved viral protein hijacks a host phosphatase to disable STAT2 signaling and preserve its antagonistic capacity, we highlight phosphorylation dynamics as a fundamental battleground in virus‐host conflict. The conservation of ORF9b within sarbecoviruses, its accumulation during infection and its presence in virions emphasize its evolutionary role as a driver of zoonotic adaptation and pandemic emergence. Beyond coronaviruses, our findings illuminate a broader principle whereby pathogens hijack host phosphatases to disarm innate immunity. Targeting host phosphatase activity may circumvent viral mutational escape, offering durable and broad‐spectrum antiviral strategies. Thus, the ORF9b‐PPM1A axis not only enriches our understanding of coronavirus pathogenesis but also exemplifies how dissecting viral exploitation of host signaling can inspire innovative interventions against current and future viral threats.

## 4 Methods

### 4.1 Cell Lines

HEK293T (CRL‐3216), A549 (CCL‐185), and HeLa (CCL‐2) cells were obtained from the American Type Culture Collection (ATCC). Huh7 (JCRB0403) cells were obtained from the Japanese Collection of Research Bioresources (JCRB). Human ACE2‐expressing HEK293T cells (HEK293T‐hACE2) were generated via lentiviral transduction, and the monoclonal positive cell was obtained by fluorescence‐activated cell sorting (FACS). All these adherent cells were maintained in Dulbecco's modified Eagle medium (DMEM) supplemented with 10% fetal bovine serum (FBS, Excell) and 100 U mL^−1^ penicillin‐streptomycin (Gibco). These cells were cultured at 37°C with 5% CO_2_. Only cells with passage numbers below 20 were used in experiments to ensure phenotypic stability. FreeStyle 293‐F cells (Gibco, R79007) were cultured in serum‐free Union 293F medium supplemented with 100 U mL^−1^ penicillin‐streptomycin and 2 mm L‐glutamine (Gibco), and maintained on an orbital shaker at 37°C with 8% CO_2_.

### 4.2 Microbe Strains

*Escherichia coli* (*E. coli*) DH5α (AlpalifeBio) was used for molecular cloning and plasmid amplification. *E. coli* BL21 (DE3) (AlpalifeBio) was used for recombinant protein expression. All bacterial strains were cultured in Luria‐Bertani (LB) medium supplemented with 100 mg L^−1^ ampicillin or 50 mg L^−1^ kanamycin at 37°C.

### 4.3 Viruses

SARS‐CoV‐2 original strain Wuhan‐Hu‐1 and Omicron sublineage JN.1 were obtained from the Guangdong Provincial Center for Disease Control and Prevention and propagated in Vero E6 cells. All authentic manipulations involving SARS‐CoV‐2 (Wuhan‐Hu‐1) and SARS‐CoV‐2 (JN.1) were carried out under Biosafety Level 3 (BSL‐3) conditions with positive‐pressure respirators at Guangzhou National Laboratory, with approval from the institutional Biosafety Committee (Approval Number: GZLAB‐AUCP‐2025‐06‐A03). Experiments involving SARS‐CoV strain GZ50 were conducted under the BSL‐3 facility at the Department of Microbiology, The University of Hong Kong. Vesicular stomatitis virus (VSV) and Sendai virus (SeV) were kindly provided by Prof. Shaobo Wang from Guangzhou National Laboratory. All VSV‐ and SeV‐related experiments were conducted at the BSL‐2 facility of Guangzhou National Laboratory.

### 4.4 Animals

K18‐hACE2 transgenic mice [C57BL/6JGpt‐*H11^em1Cin(K18‐hACE2)^*/Gpt] (strain no.: T037657) were purchased from GemPharmatech Co., Ltd. (Nanjing, China) and maintained under specific pathogen‐free (SPF) conditions. All experimental protocols involving animals were carried out in strict compliance with the guidelines of ARRIVE and were approved by the Ethics Committee of Guangzhou National Laboratory (Approval Number: GZLAB‐AUCP‐2023‐03‐A05). All SARS‐CoV‐2‐related procedures, including mouse challenge, euthanasia, tissue harvesting, and virus titration, were carried out under ABSL‐3 conditions at Guangzhou National Laboratory, in strict adherence to institutional, national, and international standards for animal welfare.

### 4.5 Coronavirus Genomes and Phylogenetic Analyses

Coronavirus genome sequences were retrieved from public databases to investigate the evolutionary distribution of ORF9b. Available coronavirus genomes with minimum genome lengths of 20 kb, excluding SARS‐CoV‐2, were downloaded from the NCBI Virus database (*n* = 8,091, accessed November 24, 2025). Eighteen SARS‐CoV‐2 reference and variant genomes, including Wuhan‐Hu‐1 and representative Alpha, Beta, Gamma, Delta, and Omicron sublineages (B.1.1.529, BA.1, BA.4, BA.5, BA.2.75, BQ.1.1, XBB.1.5, XBB.1.16, EG.5, BA.2.86, JN.1, XFG.2, and KP.3), were obtained from the GISAID database. To identify ORF9b homologs across coronaviruses, a local nucleotide database was constructed from above downloaded coronavirus genomes. The SARS‐CoV‐2 (Wuhan‐Hu‐1) ORF9b protein sequence was used as a query in tblastn (BLAST+ 2.17.0) with the following parameters: ‐evalue 1e‐5 ‐seg no ‐max_target_seqs 20000 ‐outfmt 6. Significant hits were extracted and corresponding genomic regions were retrieved for downstream analyses. To determine the phylogenetic placement of ORF9b‐positive viruses, amino acid sequences of the canonical structural proteins spike (S), envelope (E), membrane (M), and nucleocapsid (N) from the identified accessions, together with representative coronavirus sequences, were aligned and used to construct phylogenetic trees. Multiple sequence alignments were performed using MAFFT (–auto, version 7.525), and phylogenetic trees were reconstructed with the maximum likelihood method implemented in IQ‐TREE (version 3.0.1) using default parameters. Tree visualization and annotation were carried out using iTOL. For conservation analysis, ORF9b sequences with query coverage ≥ 88% were extracted and multiple sequence alignments were performed using MAFFT (–auto, v7.525), and sequence logos were generated with WebLogo 3. Sequence similarity of ORF9b at both the protein and nucleotide levels was analyzed using the seqinr package in R (version 4.4.3).

### 4.6 Plasmid Construction

*ORF9b* DNA sequences from SARS‐CoV‐2 (Wuhan‐Hu‐1), SARS‐CoV (Tor2), bat coronavirus RaTG13, civet SARS CoV 007/2004, and pangolin coronavirus MP789 were codon‐optimized (GenScript) and synthesized (Qingke Biotechnology). The constructs were designated as ORF9b (SARS2), ORF9b (SARS), ORF9b (Bat), ORF9b (Civet), and ORF9b (Pangolin), respectively. *ORF9b* genes were cloned into pcDNA3.1 vectors with a C‐terminal HA tag for mammalian expression or into pET28a with a C‐terminal 6×His tag for bacterial expression. *ORF9b* DNA sequences from additional sarbecoviruses, including bat SARS CoV Rp3/2004, bat coronavirus Rp/Shaanxi2011, bat coronavirus Cp/Yunnan2011, and BtRs‐BetaCoV/GX2013 were synthesized as described above. The corresponding constructs were designated as ORF9b (Rp3), ORF9b (SX2011), ORF9b (YN2011), and ORF9b (GX2013), respectively. The DNA sequences of *PPM1A*, *PPM1B*, and *PPP2CA* genes were obtained from cDNAs of A549 cells and were cloned into pcDNA3.1 with a C‐terminal Flag tag. The PPM1A (D239N) phosphatase‐dead mutant was generated by site‐directed mutagenesis and verified by Sanger sequencing.

### 4.7 Western Blot

A549, HEK293T, HEK293T‐sgNT, HEK293T‐sgPPM1A, A549‐sgNT, or A549‐sgPPM1A cells were seeded in 12‐well plates and transfected at 70–80% confluence with sarbecovirus ORF9b‐expressing or indicated plasmids using Lipofectamine 2000 (Invitrogen). At 18–24 h post‐transfection (h.p.t.), cells were infected with VSV at a multiplicity of infection (MOI) of 0.1 or stimulation with RO8191 (TargetMol, 10 µm) or recombinant IFN‐β (Sino Biological, 100 ng mL^−1^) for indicated time points. Harvested cells were lysed in NP‐40 lysis buffer (10 mm Tris‐HCl, 150 mm NaCl, 0.5% NP‐40, 1% Triton X‐100, 10% glycerol, 2 mm EDTA, 1 mm NaF, and 1 mm Na_3_VO_4_, pH 7.5) supplemented with phosphatase inhibitor cocktail (Fdbio science) and protease inhibitor cocktail (TargetMol) for 30 minutes (min) on ice. Supernatants were collected by centrifugation, mixed with protein loading buffer, and boiled prior to electrophoresis. Samples were separated on 4–20% or 4–12% gradient SDS‐PAGE gels (GenScript) and subjected to western blot using antibodies against TBK1 (CST, 3504), p‐TBK1 (CST, 5483), IRF3 (CST, 11904), p‐IRF3 (CST, 4947), STAT1 (CST, 14994), p‐STAT1 (Tyr701) (Selleck, F0199), STAT2 (Proteintech, 16674‐1‐AP), p‐STAT2 (Tyr690) (CST, 88410), Flag (MBL, PM020, rabbit antibody), Flag (MBL, M185‐3, mouse antibody), HA (MBL, M180‐3), IFN‐β (CST, 73671), ISG15 (Proteintech, 15981‐1‐AP), ISG56 (CST, 14769), and IFITM1 (Proteintech, 60074‐1‐Ig). GAPDH (Proteintech, 10494‐1‐AP) was used as a loading control. Imaging was performed using Odyssey M imaging system (LI‐COR Biosciences). For densitometric quantification, band intensities were measured using ImageJ software. The densitometric ratio of phosphorylated protein to total protein was calculated for each sample. For time‐course experiments, ratios were normalized to the 30‐min time point within each biological replicate.

### 4.8 Immunofluorescence Assay

For co‐localization analysis via immunofluorescence (IF) assay, HEK293T cells were seeded in six‐well plates with cover glasses which were pretreated with poly‐lysine (poly K). Cells were co‐transfected with HA‐tagged sarbecovirus ORF9b and PPM1A‐RFP, TOM70‐RFP, or other plasmids. Twenty‐four hours (h) later, cells were fixed with 4% paraformaldehyde and permeabilized with PBS supplemented with 0.2% Triton X‐100. Cells were further blocked with 5% BSA for 1 h at room temperature. Subsequently, cells were sequentially incubated with anti‐HA antibodies (MBL, M180‐3) and fluorescently labeled secondary antibodies. After each antibody incubation, cells were washed five times with PBST, ten min per wash. Nuclei were stained with 4',6‐diamidino‐2‐phenylindole (DAPI) (Invitrogen), and coverslips were mounted using the ProLong Diamond Antifade Mountant (Invitrogen) to preserve fluorescence. Imaging was performed using a NIKON N‐SIM super‐resolution microscope. For IRF3 nuclear translocation analysis, HEK293T cells were co‐transfected with indicated sarbecovirus ORF9b plasmids or empty vector and IRF3‐RFP for 24 h, followed by stimulation with poly(I:C) for 8 h. Cells were then fixed and imaged as described above. For STAT2 nuclear translocation analysis, HEK293T cells were co‐transfected with STAT2‐GFP and PPM1A‐RFP‐ or ORF9b (SARS2)‐expressing plasmids, or with empty vector. At 24 h.p.t., cells were stimulated with RO8191 (10 µm) for 8 h. Cells were then fixed and imaged as described above. Nuclear accumulation of STAT2‐GFP was assessed by fluorescence microscopy.

### 4.9 Dual‐Luciferase Reporter Assay

HEK293T cells were seeded in 12‐well plates at 12–24 h prior to transfection. Sarbecovirus ORF9b‐expressing plasmids were transfected into cells together with 100 ng of luciferase‐expressing reporter plasmids (either IFN‐β‐Luc or ISRE‐Luc) and 10 ng of *Renilla* luciferase control plasmids (pRL‐TK). At 24 h.p.t., cells were either infected with VSV or stimulated with RO8191 (10 µm) for 12 h. Cells were then washed twice with PBS and lysed in harvesting buffer (50 mm Tris‐HCl, 1 mm DTT, and 0.1% Triton X‐100, pH 7.5) for 5 min on ice. Luciferase activities were measured using a Dual Luciferase Reporter Gene Assay Kit (YEASON) according to the manufacturer's instructions.

### 4.10 Spatial Proximity‐Induced Direct Enzymatic Reporter (SPIDER) Assay

The streptavidin (SA) and Pup^E^ fusion construct (SA‐Pup^E^) was generated by polymerase chain reaction (PCR) and cloned into the pET28a expression vector. The PafA gene was cloned into the pTrc99a vector. C‐terminal His‐ and Avi‐tagged (GLNDIFEAQKIEWHE) ORF9b (SARS2) was cloned into pET32a, and the resulting plasmid was co‐transformed with pET28a‐BirA into *E. coli* BL21(DE3) to express and purify biotinylated ORF9b (SARS2) proteins. HEK293T cells were lysed in M‐PER mammalian protein extraction reagent (Thermo Scientific). For the SPIDER reaction, biotinylated ORF9b (SARS2) protein (2.5 µm) was mixed with prey cell lysate (3 mg total proteins) in reaction buffer (50 mm Tris‐HCl, 150 mm NaCl, 20 mm MgCl_2_, and 10% glycerol) and reacted for 120 min at 4°C with gentle rotation, followed by the addition of PafA (1 µm) and further incubation for 30 min with rotation. Subsequently, ATP (10 mm) and SA‐Pup^E^ (10 µm) were added, and the mixture was further incubated at 30°C for 30 min. To remove non‐covalently associated proteins, urea (8 M) was added to the reaction and incubated at 37°C for 5 min. Biotin‐agarose beads (Sigma‐Aldrich) were then added to the sample and incubated overnight at 4°C with rotation to enrich covalently pupylated prey proteins. After extensive washing, captured proteins on beads were reduced with 10 mM dithiothreitol (DTT) at 37°C for 1 h and alkylated with 25 mm iodoacetamide (IAA) in the dark for 20 min. After rinsing the beads with 200 µL of 50 mm ammonium bicarbonate (NH_4_HCO_3_), proteins were digested overnight with trypsin (Promega) at 37°C. The resulting peptides were desalted and analyzed on a nanoElute 2 system coupled to a timsTOF Pro 2 mass spectrometer (Bruker Daltonics). Raw files were processed using Spectronaut (version 20.4, Biognosys).

### 4.11 Co‐Immunoprecipitation (Co‐IP) Assay

HEK293T cells were seeded in 6‐cm dishes and co‐transfected with indicated plasmids. At 48 h.p.t., cells were gently washed twice with PBS and a cell scraper was used to detach adherent cells, followed by centrifugation to pellet cells. Subsequently, cells were lysed in 600 µL of NP‐40 lysis buffer on ice for 30 min. About 80 µL of the supernatant was collected as the total fraction, which was mixed with protein loading buffer and boiled at 100°C. The remaining lysate was incubated with anti‐Flag (Sigma‐Aldrich) or anti‐HA (Sigma‐Aldrich) resins overnight at 4°C. The resins were washed at least four times with STN washing buffer (10 mm Tris‐HCl, 150 mm NaCl, 0.5% NP‐40, and 0.5% TritonX‐100, pH 7.5). Bound proteins were eluted with 80 µL of NP‐40 lysis buffer, which were boiled with loading buffer. Both total and IP fractions were separated by 4–20% gradient SDS‐PAGE gels and analyzed by western blot using antibodies against HA (MBL, M180‐3), Flag (MBL, PM020), and GAPDH (Proteintech, 10494‐1‐AP).

### 4.12 Co‐IP‐MS Assay

HeLa cells in 6‐cm dishes were transfected with HA‐tagged ORF9b (SARS2) or empty vector plasmids and cultured for 48 h, followed by harvesting and lysis. The clarified lysate was incubated with anti‐HA resins overnight at 4°C with rotation. Resins were washed four times with STN washing buffer, followed by washing four times with PBS to remove residual NP‐40. The cleaned resins were subsequently subjected to on‐bead digestion in 100 mm ammonium bicarbonate: an initial tryptic digestion at 25°C for 2 h, followed by 30‐min reduction with 5 mm DTT, 30‐min alkylation with 10 mm iodoacetamide, and a second tryptic digestion at 25°C for 1 h. The proteolysis was quenched with trifluoroacetic acid, and the resulting peptides were desalted using C18 StageTips prior to LC‐MS/MS analysis on an Orbitrap Exploris 480 (data‐dependent acquisition). The MS raw files were processed in Thermo Scientific Proteome Discoverer (PD) (version 2.5) using the SEQUEST HT search engine and searched against a user‐provided database combined with the UniProt human reference (20,397 entries, downloaded on 16 Oct 2022). Search parameters were set as follows: up to two missed tryptic cleavages were allowed, precursor mass tolerance of 10 ppm, fragment mass tolerance of 0.02 Da, carbamidomethylation of cysteine (C) was set as a fixed modification, while oxidation of methionine (M) and protein N‐terminal acetylation were set as variable modifications. Peptide and protein identifications were filtered at a 1% false discovery rate (FDR). The final identifications and quantitative values were exported from PD for downstream interaction scoring and statistical analysis.

### 4.13 Structural Modeling Using AlphaFold 3

The amino acid sequences of ORF9b and PPM1A were submitted to the AlphaFold 3 (AF3) server for structure prediction. To increase sampling diversity and improve model reliability, the ORF9b‐PPM1A complex was predicted 20 independent times using different random seeds. Generated models demonstrating high confidence, with predicted template modeling (pTM) and interface predicted template modeling (ipTM) scores exceeding 0.5, were included. All predicted models were downloaded and structurally inspected in PyMOL (version 3.0.4). Protein‐protein interaction interfaces were analyzed in PyMOL by examining intermolecular contacts, including hydrogen bonds, salt bridges, and hydrophobic interactions. Contact residues were defined based on a distance cutoff of ≤ 4.0 Å between heavy atoms. Interface hotspots were identified by comparing recurrent contact residues across multiple predicted models. In addition, the monomeric structures of wild‐type ORF9b, the phosphomimetic mutants S50E, S53E, and S50E/S53E, the phospho‐deficient mutants S50A, S53A, and S50A/S53A, and the phospho‐deficient octuple mutant ORF9b (p8M) were predicted using the same AF3 settings. Each mutant model was superimposed onto the wild‐type model in PyMOL (version 3.0.4), and the global Cα RMSD and the local Cα RMSD over residues within 5 Å of the mutated site (for p8M, within 5 Å of any of the eight substituted sites) were calculated. The change in folding stability upon mutation (ΔΔG) and the deviations in the backbone‐related energy terms (omega, rama_prepro, and cart_bonded) were calculated with the cartesian_ddg protocol in Rosetta using the ref2015_cart score function.

### 4.14 siRNA Transfection

To knock down (KD) PPM1A, three siRNAs targeting the human *PPM1A* (RiboBio) (siPPM1A‐1: 5'‐GAAACATGGTGCAGATAGA‐3', siPPM1A‐2:5'‐ GCTGGCGTGTTGAAATGGA‐3', siPPM1A‐3: 5'‐GTCGACACCTGTTTGTATA‐3') were co‐transfected in A549 cells using Lipofectamine RNAiMAX (Invitrogen) for 12 h. The KD efficiency was confirmed by reverse transcription‐quantitative polymerase chain reaction (RT‐qPCR) and western blot. At 12 h.p.t., ORF9b‐expressing plasmids were transfected. After an additional 24 h, cells were infected with VSV for indicated times. Cells were then harvested for protein and RNA extractions. Protein samples were subjected to 4–20% gradient SDS‐PAGE gels for western blot with indicated antibodies, while RNA samples were subjected to mRNA quantifications of *IFNB1* and ISGs.

### 4.15 CRISPR‐Cas9‐sgRNA‐Mediated Knockout

The endogenous PPM1A was knocked out (KO) in A549 and HEK293T cells using the CRISPR‐Cas9 system. Three sgRNAs targeting human PPM1A (sgPPM1A#1: 5'‐ TGCCAAGTGGACTTGAATCG‐3', sgPPM1A#2: 5'‐ TCGCTAATGTGCGCATCACA‐3', sgPPM1A#3: 5'‐ GTAATGATTCAGCGTGTGAA‐3') were designed and cloned into the lentiviral vector lentiCRISPR v2 (Addgene, 52961) following standard protocols. A non‐targeting sgRNA (sgNT: 5'‐ACGGAGGCTAAGCGTCGCAA‐3') was used as a negative control. Lentiviral particles were produced in HEK293T cells by co‐transfecting 3 µg of the VSV‐G envelope plasmid (Addgene, 12259), 6 µg of the lentiviral packaging plasmid psPAX2 (Addgene, 12260), and 6 µg of the Cas9/sgRNA‐expressing lentiCRISPR v2 constructs using PEI MAX (Polysciences). At 48 h.p.t., culture supernatants containing lentiviral particles were collected and concentrated overnight using PEG 6000. A549 and HEK293T cells were subsequently infected with sgNT or sgPPM1A lentiviruses. Following infection, cells were treated with 1 µg mL^−1^ puromycin to eliminate uninfected cells and generate stable PPM1A‐KO cell populations. The KO efficiency was confirmed by western blot with antibodies against PPM1A (CST, 3459). To test whether the immune‐antagonistic activity of ORF9b depended on PPM1A, sgPPM1A#1 and sgNT A549 cells were transfected with ORF9b (SARS2)‐expressing plasmid or empty vector, followed by VSV infection or RO8191 stimulation, and the resulting IFN/ISG expression was measured as indicated in the figure legends. For complementation experiments, PPM1A‐KO A549 cells (sgPPM1A#1) were transfected with PPM1A (WT)‐expressing plasmid or empty vector together with ORF9b (SARS2) for 24 h, followed by VSV infection or RO8191 stimulation as indicated in the figure legends. To enable authentic SARS‐CoV‐2 infection in PPM1A‐deficient cells, PPM1A‐KO (sgPPM1A#1) and non‐targeting (sgNT) HEK293T cells were transfected with a plasmid expressing human ACE2, and subsequently infected with SARS‐CoV‐2 (JN.1) in the presence of SMIP‐031 (0, 0.3125 or 0.625 µM) to determine whether its antiviral activity required PPM1A.

### 4.16 Reverse Transcription‐Quantitative Polymerase Chain Reaction (RT‐qPCR)

HEK293T cells were seeded in 12‐well plates and transfected with sarbecovirus ORF9b‐expressing plasmids. At 24 h.p.t., cells were stimulated with VSV, SeV, poly(I:C), RO8191, or recombinant IFN‐β for 12 h. Cells were then harvested for total RNA extraction using the EZ‐press RNA Purification Kit (EZBioscience) according to the manufacturer's protocol. For homogenized mouse lung tissues, RNAs were extracted via the Universal RNA Purification Kit (EZBioscience) under BSL‐3 facility. Reverse transcription (RT) was performed using 1 µg of total RNA to synthesize cDNA with the All‐in‐one RT SuperMix Perfect for qPCR Kit (Vazyme). Quantitative polymerase chain reaction (qPCR) quantifications for *IFNB1*, *ISG15*, *ISG56*, *IFITM1*, *mIsg15*, *mIsg56*, and *mIfitm1* mRNA expression were conducted using ChamQ Universal SYBR qPCR Master Mix (Vazyme) on the QuantStudio 1 Plus detection system (Applied Biosystems). The housekeeping gene *GAPDH* was used as the internal control. Relative mRNA expression levels were calculated using the 2^−ΔΔCt^ method.

### 4.17 Protein Purification

Recombinant sarbecovirus ORF9b homologs and mutants with a C‐terminal 6×His‐tag were expressed under the pET28a vector backbone. The plasmids were transformed into *E. coli* BL21 (DE3) competent cells. Single bacterial clones were selected and cultured at 37°C until the optical density at 600 nm (OD 600) reached 0.4‐0.6, followed by induction with 1 mM Isopropyl β‑D‑1‑thiogalactopyranoside (IPTG) overnight at 16°C. Bacteria were harvested by centrifugation at 9000 rpm for 3 min and resuspended in Buffer A (20 mM Tris and 50 mM NaCl, pH 7.5). Cells were then lysed using a high‐pressure homogenizer, and the lysate was clarified by centrifugation and filtration through a 0.45 µm filter. The clarified supernatant was incubated with TALON Superflow beads (Cytiva). Bound proteins were eluted with linear gradients of imidazole‐containing buffers, with concentrations of imidazole ranging from 5 to 300 mm. At each elution step, samples were analyzed by SDS‐PAGE followed by Coomassie blue staining to assess purity and yield. The purified proteins were concentrated and buffer‐exchanged with Buffer A by ultrafiltration, followed by further purification by size‐exclusion chromatography (SEC).

For the purification of His‐tagged PPM1A proteins expressed in mammalian cells, the PPM1A coding sequence was fused with an N‐terminal secretion signal peptide (MGILPSPGMPALLSLVSLLSVLLMGCVA) and a C‐terminal 6×His tag, and cloned into the pcDNA3.1 vector. The construct was transfected into FreeStyle 293‐F cells using PEI MAX (Polysciences). On day 7 post‐transfection, the culture supernatant was collected and clarified by centrifugation and filtration through a 0.45 µm filter. The supernatant was incubated with Ni Sepharose Excel agarose resins (Cytiva) for affinity purification. The target protein was eluted with linear gradients of imidazole‐containing buffers, with concentrations of imidazole ranging from 5 to 500 mm. Eluted fractions were analyzed by SDS‐PAGE, concentrated by ultrafiltration, and further purified by SEC.

For the purification of Flag‐tagged STAT2 and GFP proteins, the coding sequences of corresponding genes were cloned into the pcDNA3.1 vector with a C‐terminal Flag tag and transfected into FreeStyle 293‐F cells. On day 3 post‐transfection, cells were harvested and resuspended in Buffer A supplemented with protease inhibitor cocktail (TargetMol). Cells were lysed by sonication, and the lysate was clarified by centrifugation for 1 h, followed by filtration through a 0.45 µm filter. The filtered supernatant was incubated with anti‐Flag resins (GenScript) to enrich Flag‐tagged target proteins, followed by elution with 150 µg mL^−1^ of FLAG peptides (Sangon Biotech). Target proteins were concentrated and proceeded with SEC to remove residual FLAG peptides. All protein concentrations were determined by the BCA assay (ThermoFisher Scientific) according to the manufacturer's instructions.

### 4.18 Surface Plasmon Resonance (SPR) Assay

The binding affinities between sarbecovirus ORF9b homologs and PPM1A were measured using Biacore 8K plus system (Cytiva). Purified ORF9b proteins were diluted in 10 mm acetate buffer (pH 5.0) to about 10 µg mL^−1^ and immobilized onto a CM5 sensor chip (Cytiva) which was pre‐activated, followed by blocking the chip by ethanolamine. His‐tagged PPM1A proteins (analytes) were diluted in PBS‐P buffer at concentrations ranging from 0 to 2000 nm and injected at a constant flow rate. To measure the binding affinity between PPM1A and STAT2, STAT2 proteins were diluted in 10 mm acetate buffer (pH 5.0) to about 10 µg mL^−1^ and immobilized onto a CM5 sensor chip. His‐tagged PPM1A proteins were diluted in PBS‐P buffer, with concentrations ranging from 0 to 1000 nm, and injected as the analytes. For measuring the binding affinity between PPM1A and SMIP‐031, His‐tagged PPM1A proteins were diluted in 10 mm acetate buffer (pH 4.5) to about 10 µg mL^−1^ and were immobilized onto a CM7 sensor chip (Cytiva). SMIP‐031 compounds were diluted in 5% DMSO PBS‐P buffer at concentrations ranging from 0 to 15.625 µm and injected as the analytes. Association and dissociation kinetics were analyzed using Biacore evaluation software to determine the association rate constant (“On rate”, *k* *_a_*) and dissociation rate constant (“Off rate”, *k* *_d_*). The equilibrium dissociation constant (“Binding constant”, *K_D_*) was calculated as *k* *_d_*/*k* *_a_*.

### 4.19 In Vitro Kinase Assay and Phosphatase Assay

Recombinant ORF9b‐6His and PPM1A‐6His proteins were in vitro purified as described above. A MARK3 kinase assay kit (Promega) was used to phosphorylate ORF9b. Briefly, ORF9b proteins were co‐incubated with recombinant MARK3 in the kinase reaction buffer at room temperature for 45 min to generate phosphorylated ORF9b (phos‐ORF9b). Subsequently, the ADP‐Glo reagent was added to deplete unreacted ATP. ADP molecules, which were generated from the consumption of reacted ATP, were reconverted to ATP with the kinase detection reagent. Simultaneously, the newly generated ATP was converted to light by the luciferase reaction. Luminescence units were recorded to represent the activity of MARK3‐mediated ORF9b phosphorylation.

The in vitro PPM1A phosphatase activity assay was conducted utilizing Malachite Green Phosphate Detection Kit (Beyotime). In vitro purified ORF9b proteins were phosphorylated via the MARK3 kinase assay. Phos‐ORF9b proteins were then concentrated and buffer‐exchanged with PBS via ultrafiltration. Recombinant PPM1A and phos‐ORF9b proteins were co‐incubated in the phosphatase buffer, facilitating PPM1A to dephosphorylate phos‐ORF9b. The released phosphates were subsequently detected with the malachite green phosphate detection reagent. Molybdates reacted with phosphates to form a phosphomolybdate complex that interacted with malachite green, producing a green to blue‐green colored complex. The color intensity, which was measured by absorbance at 630 nm, was proportional to the amounts of phosphates released by the phosphatase reaction.

For the in vitro STAT2 phosphatase assay, HEK293T cells were overexpressed with STAT2‐Flag for 48 h and stimulated with RO8191 (10 µm) for 8 h. Cells were harvested and lysed, and the lysate was incubated with anti‐Flag resin for 4 h at 4°C with rotation. Bound proteins were eluted with 3×Flag peptides, concentrated by ultrafiltration, and incubated with purified recombinant PPM1A‐6His in phosphatase buffer for 45 min at room temperature. The phosphorylation of STAT2 at Tyr690 was assessed by western blot using an anti‐p‐STAT2 (Tyr690) antibody (CST, 88410T).

### 4.20 Pull‐Down Assay

Recombinant PPM1A‐6His, STAT2‐Flag, and GFP‐Flag proteins were expressed in FreeStyle 293‐F cells as described above. STAT2‐Flag or GFP‐Flag proteins were co‐incubated with PPM1A‐6His at 4°C overnight in the presence of anti‐Flag beads. For the ORF9b‐PPM1A‐STAT2 ternary complex pull‐down, STAT2‐Flag or GFP‐Flag proteins were co‐incubated with PPM1A‐6His and ORF9b (SARS2)‐6His at 4°C for 4 h in the presence of anti‐Flag beads. Following incubation, beads were washed at least four times with STN wash buffer to remove non‐specifically bound proteins. Bound proteins were eluted with NP‐40 lysis buffer and boiled with loading buffer. The samples were separated by 4–20% SDS‐PAGE gel and analyzed by both Coomassie staining and western blot.

### 4.21 Transmission Electron Microscopy

Vero E6 cells were infected with authentic SARS‐CoV‐2 at MOI of 0.1 for 3 d. Culture supernatants were collected and fixed with 1% formaldehyde overnight at room temperature. Viral particles were concentrated by ultracentrifugation through a 30% sucrose cushion at 42 000 × *g* for 120 min at 4°C in a Sorvall WX100+ ultracentrifuge using a T‐865 rotor (ThermoFisher Scientific). The pelleted particles were resuspended with MOPS‐saline‐EDTA (MSE) buffer (10 mm MOPS, 150 mm NaCl, and 1 mm EDTA, pH 6.8). For negative staining, 5 µL of viral suspensions were applied onto carbon‐coated copper grids and allowed to adsorb for 10 min, followed by blotting with filter paper and negatively staining with 2% (w/v) uranyl acetate for 1 min. Excess stain was removed, and the grids were air‐dried. Samples were then examined using the transmission electron microscope (TEM) operated at an accelerating voltage of 120 kV (Talos L120C, ThermoFisher Scientific). Images were captured at a magnification of 11,000× with a nominal defocus of ‐2.4 µm.

For immunogold electron microscopy (iEM) analysis of SARS‐CoV‐2 virions, viral suspensions were applied to nickel grids, followed by quenching with 0.05 m glycine, permeabilization with 0.1% Triton X‐100, and blocking with 5% BSA. After each step, grids were washed five times with PBS (3 min per wash). Grids were then incubated overnight at 4°C with primary antibodies against SARS‐CoV‐2 N (Sino Biological, 40143‐R001) or ORF9b (GeneTex, GTX637667‐S) (1:100 dilution), followed by incubation with 6‐nm gold‐conjugated goat anti‐rabbit IgG (Jackson ImmunoResearch, 111‐195‐144) (1:100 dilution) for 1.5 h at room temperature. After primary and secondary antibody incubations, grids were washed eight times with PBS (3 min per wash). Samples were fixed with 2.5% glutaraldehyde, negatively stained with 2% (w/v) uranyl acetate, and imaged at 36,000× magnification with a nominal defocus of ‐2.4 µm using a Talos L120C transmission electron microscope operated at 120 kV.

### 4.22 Authentic Virus Infection Assay

To investigate the influence of ORF9b (SARS2), PPM1A, and MARK3 on authentic SARS‐CoV‐2 replication and innate immune responses, HEK293T‐hACE2 cells in 24‐well plates were overexpressed with increasing amounts of ORF9b (SARS2), PPM1A, and MARK3 for 24 h, respectively, followed by infection with 1 MOI of SARS‐CoV‐2 (Wuhan‐Hu‐1) or SARS‐CoV‐2 (JN.1) for 12 h or 24 h. Total RNAs were extracted via the EZ‐press RNA Purification Kit (EZBioscience). Viral RNA copies were quantified by RT‐qPCR targeting the SARS‐CoV‐2 *N* gene (SARS2‐N‐F: 5'‐CAGTAGGGGAACTTCTCCTGCT‐3', SARS2‐N‐R: 5'‐TCTGTCAAGCAGCAGCAAAG‐3', SARS2‐N‐Probe: 5'‐FAM‐CTGGCAATGGCGGTGATGCTGC‐BHQ1‐3'), using the one‐step SARS‐CoV‐2 RNA detection kit (Daan Gene, DA0992). RT‐qPCR quantifications of *IFNB1*, *ISG15*, *ISG56*, and *PPM1A* were conducted using ChamQ Universal SYBR qPCR Master Mix (Vazyme) as described above. To investigate the influence of PPM1A inhibitor SMIP‐031 and MARK3 inhibitor PCC0208017 on SARS‐CoV‐2 replication, HEK293T‐hACE2 cells in 24‐well plates were co‐cultured with indicated concentrations of compounds and authentic viruses for 24 h. Viral RNA copies were quantified as above.

To verify the effects of ORF9b (SARS), PPM1A, and MARK3 on authentic SARS‐CoV replication, HEK293T‐hACE2 cells in 24‐well plates were overexpressed with increasing amounts of these proteins, respectively. At 24 h.p.t., cells were infected with 1 MOI of SARS‐CoV (GZ50) for 16 h. The mRNA expression levels of intracellular SARS‐CoV *N* gene were quantified by RT‐qPCR (SARS‐N‐F: 5'‐ACCAGAATGGAGGACGCAATG‐3', SARS‐N‐R: 5'‐GCTGTGAACCAAGACGCAGTATTAT‐3') and normalized to those of *GAPDH*. The effects of SMIP‐031 and PCC0208017 on SARS‐CoV replication were conducted in Huh7 cells. Huh7 cells in 24‐well plates were co‐incubated with indicated concentrations of each compound and authentic viruses for 24 h, followed by the RT‐qPCR quantification of viral RNAs.

### 4.23 Mice Infection Assay

To evaluate whether prophylactic administration of the PPM1A inhibitor SMIP‐031 protects against SARS‐CoV‐2 infection in vivo, K18‐hACE2 transgenic mice aged 4–6 weeks were randomly assigned into three groups (*n* = 5 per group): a control group intraperitoneally (i.p.) administered with an equal volume of vehicle (10% DMSO, 30% PEG300, 5% Tween‐80, and 55% PBS), and two treatment groups receiving SMIP‐031 at 10 and 15 mg kg^−1^, respectively. At 4 h post‐treatment, all mice were intranasally (i.n.) challenged with 1×10^5^ focus‐forming units (FFUs) of authentic SARS‐CoV‐2 (JN.1), followed by continuous treatments with SMIP‐031 at 8, 24, 36, and 48 h after the primary treatment. Mice were euthanized at 6 h after the final administration, and lung tissues were collected for subsequent analysis. One lung lobe of each mouse was fixed in 4% paraformaldehyde for hematoxylin and eosin (H&E) staining and immunohistochemistry (IHC) with SARS‐CoV‐2 N antibodies, while the remaining lung tissues were homogenized for RNA extraction. Viral RNA copies within RNA samples were quantified using the one‐step SARS‐CoV‐2 RNA detection kit as above, while mRNA expression levels of *mIsg15*, *mIsg56*, and *mIfitm1* in different groups were assessed by RT‐qPCR.

For quantitative histopathological scoring, lung tissue sections subjected to H&E staining were examined under light microscopy. Semi‐quantitative grading was performed by three independent observers who were blinded to treatment allocation. The scoring criteria incorporated both tissue injury (alveolar wall thickening and airspace consolidation) and inflammatory infiltration (perivascular, peribronchiolar, and interstitial) across the lung parenchyma. A 0–4 scoring system was adopted: 0, within normal limits; 1, minimal and localized alteration; 2, mild but clearly identifiable lesions; 3, moderate and multifocal pathology; 4, severe and diffusely distributed damage. The raw pathology score for each specimen was presented as a semi‐quantitative heatmap of three independent readings. For each mouse, the scores from the three observers were then averaged to yield a single score per animal, and group comparisons were performed at the animal level using the Kruskal‐Wallis test followed by Dunn's multiple comparisons tests, which are appropriate for ordinal data.

For IHC signal quantification, deparaffinized and rehydrated lung sections were subjected to antigen retrieval, blocked, and incubated with anti‐SARS‐CoV‐2 N protein antibody, followed by HRP‐conjugated secondary antibody and DAB development. IHC images were acquired under uniform illumination settings with the operator blinded to experimental groups. For each mouse, three randomly selected fields of the lung sections were imaged, and the integrated density of DAB‐positive area was quantified by color deconvolution in ImageJ and averaged across the three fields to yield a single value per animal. Statistical comparisons for IHC quantification were performed using one‐way ANOVA followed by Dunnett's multiple comparisons tests, as appropriate for the data distribution.

### 4.24 Statistics

All statistical analyses were performed in GraphPad Prism 9.5 and R software. Data in graphs were presented as the mean ± standard errors of the mean (SEM) from at least three independent biological experiments. Statistical comparisons were performed using Student's *t*‐test, one‐way ANOVA, two‐way ANOVA, or Kruskal‐Wallis test as appropriate. A *P* value less than 0.05 was considered statistically significant. Statistical significances were denoted as follows: *P < 0.05, **P < 0.01, and ***P < 0.001.

## Author Contributions

Conceptualization, L.X., Y.H., W.Y., H.J., and X.M.; methodology, L.X., Z.H., Z.Z., Y.Z., X.L., T.L., J.L., Y.L., Y.H., and X.M.; investigation, L.X., Z.H., Z.Z., Y.Z., X.L., L.W., T.B., T.L., J.L., M.H., G.Q., S.X., S.L., Y.L., H.R., T.C., H.P., B.Z., J.T., Q.L., Y.L., Y.H.; Writing – original draft, L.X., and X.M.; Writing – review & editing, L.X., Y.H., W.Y., H.J., and X.M.; funding acquisition, X.M.; resources, Y.L., Y.H., W.Y., H.J., and X.M.; supervision, Y.H., W.Y., H.J., and X.M.

## Conflicts of Interest

The authors declare no conflicts of interest.

## Supporting information




**Supporting File 1**: advs78023‐sup‐0001‐SuppMat.docx.

**Supporting File 2**: advs78023‐sup‐0002‐TableS1.xlsx.

## Data Availability

The mass spectrometry proteomics data have been deposited to the ProteomeXchange Consortium (https://proteomecentral.proteomexchange.org) via the iProX partner repository with the dataset identifier PXD074794 and PXD075386. A publicly available dataset deposited in the ProteomeXchange under the identifier PXD018117 was downloaded for reanalysis. The RNA‐seq data used in this study were obtained from a publicly available dataset deposited in the Sequence Read Archive (SRA) under the accession number PRJNA660067 and were downloaded for reanalysis.
