# Supplementary material for: Sarbecoviruses Suppress Innate Immunity by Recruiting PPM1A to Modulate STAT2 and ORF9b Phosphorylation
Source: Adv Sci (Weinh). 2026 Sep 27:e78023. Online ahead of print. doi: 10.1002/advs.78023 (PMC13616384; doi:10.1002/advs.78023)
Supplement: Supplementary file 1 [file ADVS-9999-e78023-s001.docx]

Supporting Information

Sarbecoviruses suppress innate immunity by recruiting PPM1A to modulate STAT2 and ORF9b phosphorylation

Lixiang Xie, Ziye Huang, Zhiyuan Zhang, Yiqiang Zhu, Xiaoqing Liu, Lei Wang, Tongyu Bi, Taizhen Liang, Jintao Lai, Meilin Hu, Guochang Qiu, Shiqi Xiao, Sen Liu, Yaoming Liu, Haiyue Rao, Tao Chen, Haojie Peng, Bin Zhang, Jia Tang, Qianying Li, Yaxin Li, Yuxin Hou,^*^ Weibo Yang,^*^ Hewei Jiang,^*^ and Xiancai Ma^*^

**Figure S1. Nucleotide and amino acid sequence conservation of ORF9b across representative sarbecoviruses.**

(**A**) Scatter plot of the 668 ORF9b homologous hits identified by tblastn, showing query coverage (%) on the y-axis versus sequence identity (%) on the x-axis. Each point represents one significant hit (E-value < 1×10^-5^). Identity ranged from 40.2% to 100%, and query coverage ranged from 51.5% to 100%.

(**B**) Heatmap showing pairwise similarity matrices of representative sarbecovirus ORF9b proteins, including those from SARS coronavirus Tor2, bat SARS CoV Rp3/2004, civet SARS CoV 007/2004, bat coronavirus Rp/Shaanxi2011, bat coronavirus Cp/Yunnan2011, BtRs-BetaCoV/GX2013, bat coronavirus RaTG13, pangolin coronavirus MP789, SARS-CoV-2_Wuhan-Hu-1, SARS-CoV-2_Alpha, SARS-CoV-2_Beta, SARS-CoV-2_Gamma, SARS-CoV-2_Delta, and SARS-CoV-2_Omicron. Amino acid and nucleotide sequence similarities were displayed in the lower and upper triangles, respectively. Values indicated percentages of sequence identity. Higher percent identities were colored in blue, while lower identities were colored in green.

(**C**) Multiple sequence alignments of representative sarbecovirus ORF9b proteins were performed using MAFFT and visualized with Jalview. In addition to protein sequences analyzed in (**A**), additional SARS-CoV-2 Omicron sublineages, namely B.1.1.529, BA.1, BA.4, BA.5, BA.2.75, BQ.1.1, XBB.1.5, XBB.1.16, EG.5, BA.2.86, JN.1, XFG.2, and KP.3, were also included. Conserved residues were highlighted in blue.

**Figure S2. ORF9b suppresses *IFNB1* and ISG expression in response to multiple innate immune stimuli.**

(**A-C**) HEK293T cells were overexpressed with ORF9b (SARS2) for 24 h, followed by infection with VSV for 12 h. RT-qPCR assays were performed to quantify mRNA expression levels of *IFNB1* (**A**), *ISG15* (**B**), and *ISG56* (**C**).

(**D-F**) The mRNA expression levels of *IFNB1* (**D**), *ISG15* (**E**), and *ISG56* (**F**) were measured as in (**A-C**), except that cells were infected with SeV for 12 h.

(**G-I**) The mRNA expression levels of *IFNB1* (**G**), *ISG15* (**H**), and *ISG56* (**I**) were measured as in (**A-C**), except that cells were stimulated with poly (I:C) for 12 h.

(**J-L**) RT-qPCR analysis of *IFNB1* (**J**), *ISG15* (**K**)**,** and *ISG56* (**L**) mRNA expression levels in HEK293T cells overexpressing ORF9b homologs from additional bat sarbecovirus isolates, followed by VSV infection.

(**M-O**) RT-qPCR analysis of *IFNB1* (**M**), *ISG15* (**N**), and *ISG56* (**O**) mRNA expression levels in HEK293T cells overexpressing ORF9b variants from the SARS-CoV-2 Wuhan-Hu-1, Omicron JN.1 isolate, or indicated mutants (P10S substitution and ENA/EDA deletion), followed by VSV infection. Western blot analysis of the corresponding ORF9b variant protein levels in parallel samples was shown below each RT-qPCR panel. GAPDH served as a loading control.

Data in (**A-O**) were presented as mean ± SEM in biological triplicates. *P* values in (**A-I**) were calculated by two-way ANOVA with Šidák's multiple comparisons tests. *P* values in (**J-O**) were calculated by one-way ANOVA with Dunnett's multiple comparisons tests. *** *P* < 0.001.

**Figure S3. ORF9b does not interact with key RIG-I/MAVS signaling components.**

(**A-E**) HA-tagged ORF9b (SARS2) was co-overexpressed with Flag-tagged RIG-I (**A**), MDA5 (**B**), MAVS (**C**), TBK1 (**D**), and IRF3 (**E**), respectively, followed by anti-HA co-immunoprecipitation (Co-IP) assays.

(**F**) Uncropped full membrane images of the western blots corresponding to the data presented in Figure 3L. Red boxes indicated the cropped regions shown in the respective figure panels. Molecular weight markers (kDa) were indicated in each membrane.

**Figure S4. Identification of ORF9b-interacting proteins by proteomics.**

(**A**) Schematic of SPIDER-MS. Briefly, Avi-tagged ORF9b (SARS2) proteins (bait) were biotinylated with BirA, followed by incubation with HEK293T prey cell lysates. The streptavidin (SA) and Pup^E^ in-frame proteins (SA-Pup^E^) and recombinant PafA proteins were subsequently added into cell lysates to facilitate proximal pupylation of ORF9b-interacting prey proteins. Biotin-agarose beads were then added to the mixtures to pull down enriched proteins, followed by on-bead digestion and subsequent MS analysis.

(**B**) Schematic of Co-IP-MS. HA-tagged ORF9b (SARS2) proteins were overexpressed in HeLa cells, followed by cell lysis at 48 hours post-transfection (h.p.t.). Anti-HA agarose beads were added into the lysates to enrich co-immunoprecipitated proteins, followed by on-bead digestion and MS identification.

(**C**) Gene set enrichment analysis (GSEA) of proteins identified by Co-IP-MS and SPIDER-MS. Reactome pathways associated with SARS-CoV and SARS-CoV-2 signaling with FDR (false discovery rate) < 0.25 are shown. No pathways from the Strep-AP-MS dataset passed this threshold and were therefore excluded from visualization.

(**D**) Protein-protein interaction network of ORF9b-interacting proteins. Proteins included in the network were derived from the union of pairwise intersections among upregulated proteins identified in three independent datasets (Strep-AP-MS, Co-IP-MS, and SPIDER-MS), yielding a total of 63 proteins. STRING analysis was subsequently performed to construct the PPI network with default parameters. Six proteins (LIN7C, RHOC, NFYC, PDE4D, EIF2A, and ARFAP2) did not form interactions within the network and were therefore excluded from visualization. The remaining 57 proteins were used for PPI network construction. Nodes represent individual proteins, and edges represent detected interactions between proteins. Node size corresponds to node popularity (degree centrality), reflecting the number of connections. Nodes were classified as High (degree > 5) or Low (degree ≤ 5), and are colored accordingly. Edge color indicates interaction strength, with Strong (value > 0.8) and Weak (value ≤ 0.8) interactions.

(**E-H**) Expression of *PPP2CA* (**E**), *PPM1G* (**F**), *PPP1CA* (**G**), and *PPP3CA* (**H**) in clinical samples. Violin plots depicted mRNA expression levels in healthy, mild, and severe COVID-19 individuals.

(**I-L**) Correlation analysis between *PPP2CA* (**I**), *PPM1G* (**J**), *PPP1CA* (**K**), and *PPP3CA* (**L**) expression and *ISG15* expression in patient samples. Linear regression analysis was performed to evaluate the association between *ISG15* and *PPP2CA*, *PPM1G*, *PPP1CA*, or *PPP3CA*. Pearson correlation coefficients (*R*) and corresponding two-sided *P* values were calculated.

*P* values in (**E-H**) were calculated using Kruskal-Wallis tests followed by Dunn's post hoc comparisons tests. ns, not significant. ** *P* < 0.01, *** *P* < 0.001.

**Figure S5. Conserved interaction between sarbecovirus ORF9b and host PPM1A.**

(**A**) HA-tagged ORF9b homologs were co-overexpressed with Flag-tagged TOM70, followed by anti-HA Co-IP analysis to validate their interactions.

(**B**) Co-IP assays validating the interaction between PPM1A and ORF9b homologs derived from additional bat sarbecoviruses.

(**C, D**) IF analysis of the subcellular localization and co-localization of ORF9b homologs with TOM70 (**C**) or PPM1A (**D**). Scale bar, 5 μm.

**Figure S6. Examination of non-functional mutations within ORF9b-PPM1A interface.**

(**A-C**) Co-IP assays assessing the interaction between ORF9b and the PPM1A mutants, including F4A (**A**), R33A (**B**), and S190A (**C**).

(**D**) Co-IP analysis of the interaction between ORF9b (Q18A) mutant and PPM1A.

(**E**) Co-IP analysis of ORF9b interaction with a PPM1A mutant containing eight simultaneous alanine substitutions (8M) at predicted interface residues, as well as corresponding single-site revertant mutants, including 8M-A2G, 8M-A4F, 8M-A33R, 8M-A35E, 8M-A186R, 8M-A188N, 8M-A189G, and 8M-A190S.

**Figure S7. PPM1A downregulates RO8191-induced STAT2 phosphorylation.**

(**A**) Uncropped full membrane images of the western blots corresponding to western blots presented in Figure 6E. Red boxes indicated the cropped regions shown in the respective figure panels. Molecular weight markers (kDa) were indicated in each membrane.

(**B-E**) Additional independent replicates of the experiment presented in Figure 6E-G. (**B**) and (**D**) presented western blot analysis of p-STAT2 and p-STAT1 levels in A549 cells overexpressing PPM1A for 24 h and stimulated with RO8191 for 30 min, 1 h, and 2 h. Data in (**C**) and (**E**) represented uncropped full membrane images corresponding to (**B**) and (**D**), respectively. Red boxes indicated the cropped regions shown in the respective figure panels. Molecular weight markers (kDa) were indicated on each membrane.

**Figure S8. PPM1A catalytic activity is required for the suppression of JAK-STAT signaling.**

(**A**) Triple co-localization analysis of HA-tagged ORF9b homologs, PPM1A-RFP, and STAT2-GFP. No RO8191 stimuli were added. Scale bar, 5 μm.

(**B**) STAT2-GFP was co-overexpressed with PPM1A-RFP or empty vector in HEK293T cells, followed by stimulation with RO8191 for 8 h. Nuclear translocation of STAT2 was analyzed by fluorescence microscopy. Scale bar, 10 μm.

(**C, D**) A549 cells were overexpressed with wild-type (WT) PPM1A or PPM1A (D239N) mutant for 24 h, followed by stimulation with RO8191 for 12 h. RT-qPCR analysis was performed to determine mRNA expression levels of *ISG15* (**C**) and *ISG56* (**D**).

(**E**) A549 cells overexpressing PPM1A (D239N) for 24 h and stimulated with RO8191 for 30 min, 1 h, or 2 h. Western blot analysis was conducted with antibodies against total STAT1, total STAT2, p-STAT1, and p-STAT2.

(**F**) HEK293T cells were overexpressed with STAT2-Flag for 48 h and stimulated with RO8191 for 12 h. Cells were harvested and lysed, followed by immunoprecipitation with anti-Flag resin for 4 h with rotation at 4℃. Bound proteins were eluted with 3×Flag peptides, concentrated by ultrafiltration, and analyzed by western blot using an anti-p-STAT2 (Tyr690) antibody.

(**G**) *In vitro* purified PPM1A-6His proteins were incubated with the enriched STAT2 proteins in an *in vitro* phosphatase assay, and the phosphorylation of STAT2 at Tyr690 residue was assessed by western blot using an anti-p-STAT2 (Tyr690) antibody.

(**H**) A549 cells were transfected with a mixture of three siRNAs targeting PPM1A (100 nM each) or a negative control siRNA (siNC). At 24 h post-transfection, cells were harvested. Total RNA was extracted, followed by the quantification of *PPM1A* mRNA expression by RT-qPCR.

(**I**) A549 cells were transfected as in (**H**). At 24 h post-transfection, cells were harvested and lysed. PPM1A protein levels were assessed by western blot to evaluate siRNA knockdown efficiency. GAPDH served as a loading control.

(**J**) Three PPM1A knockout A549 cell lines were generated by lentiviral transduction of lentiCRISPR v2 vectors encoding distinct sgRNAs targeting human PPM1A (sgPPM1A#1, #2, and #3). A non-targeting sgRNA (sgNT) served as a negative control. Transduced cells were selected with puromycin, and knockout efficiency was assessed by western blot using an anti-PPM1A antibody. GAPDH served as a loading control.

(**K-M**) PPM1A knockout A549 cells (sgPPM1A#1) were transfected with an empty vector or PPM1A-Flag plasmid for 24 h, followed by RO8191 stimulation for 12 h. *ISG15* (**K**), *ISG56* (**L**), and *IFITM1* (**M**) mRNA expression levels were quantified by RT-qPCR.

Data in (**C**), (**D**), (**H**), and (**K-M**) were presented as mean ± SEM in biological triplicates. *P* values were calculated by Student's *t*-tests. ** *P* < 0.01, *** *P* < 0.001.

**Figure S9. Phosphorylation state governs ORF9b immunosuppressive activity.**

(**A-G**) Structural models of SARS-CoV-2 ORF9b variants were predicted by AlphaFold 3. Each panel presented an overall cartoon representation of SARS-CoV-2 ORF9b (pink) and a magnified view of the local structural environment surrounding residues S50 and S53. The indicated residues and their substituted side chains were shown as sticks within a mesh representation, while residues within 5 Å of S50 or S53 are highlighted as sticks. Structural models were shown for wild-type SARS-CoV-2 ORF9b (**A**), the phosphomimetic mutants S50E (**B**), S53E (**C**), and S50E/S53E (**D**), and the phospho-deficient mutants S50A (**E**), S53A (**F**) and S50A/S53A (**G**). Each mutant model was superimposed onto wild-type ORF9b to calculate the Cα RMSD over the whole protein (global) and over residues within 5 Å of the mutated site (local). Folding free energy change (ΔΔG) and backbone-related energy terms (omega, rama_prepro, and cart_bonded, reported as the maximum absolute deviation, max |Δbackbone|) were calculated using the Rosetta cartesian_ddg protocol. The values of global Cα RMSD (Å), local RMSD (Å), ΔΔG (REU), and max |Δbackbone| (REU), respectively, were as follows: S50E, 0.381, 0.183, +0.823, 0.489; S53E, 0.453, 0.218, -1.374, 0.162; S50E/S53E, 0.397, 0.126, -0.007, 0.522; S50A, 0.598, 0.239, -0.396, 0.337; S53A, 0.505, 0.137, +1.636, 0.129; and S50A/S53A, 0.557, 0.198, +1.857, 0.178.

(**H-J**) HEK293T cells were overexpressed with WT or phosphomimetic ORF9b (SARS2) mutants (S50E, S53E, and S50E/S53E) for 24 h, followed by infection with VSV for 12 h. RT-qPCR assays were performed to determine mRNA expression levels of *IFNB1* (**H**), *ISG15* (**I**), and *ISG56* (**J**).

(**K-M**) HEK293T cells were overexpressed with WT or phospho-deficient ORF9b (SARS2) mutants (S50A, S53A, and S50A/S53A), followed by infection with VSV, and mRNA quantification of *IFNB1* (**K**), *ISG15* (**L**), and *ISG56* (**M**) was conducted as in (**H-J**).

Data in (**H-M**) were presented as mean ± SEM in biological triplicates. *P* values in (**H-M**) were calculated by one-way ANOVA with Dunnett's multiple comparisons tests. ** *P* < 0.01, *** *P* < 0.001.

**Figure S10. MARK3 is identified as an ORF9b-interacting kinase.**

(**A, B**) Co-localization of PPM1A-RFP with phosphomimetic or phospho-deficient ORF9b (SARS2) mutants was analyzed by IF. Scale bar, 5 μm.

(**C**) Co-IP assay examining the potential interaction between ORF9b (SARS2) and MARK1. No detectable interaction was observed.

(**D**) Co-IP assay examining the potential interaction between ORF9b (SARS2) and GSK3A. No detectable interaction was observed.

(**E**) Schematic of *in vitro* MARK3 kinase activity assay. Recombinant MARK3 and ORF9b proteins were co-incubated in the kinase reaction reagent to generate phosphorylated ORF9b (phos-ORF9b). The ADP-Glo^TM^ reagent was then added to deplete redundant ATP. The reacted ADP was reconverted to ATP with the kinase detection reagent. Simultaneously, the newly generated ATP was converted to light by the luciferase reaction, during which luminescence units were recorded to represent the activity of MARK3-mediated ORF9b phosphorylation.

(**F**) The phospho-deficient ORF9b (p8M), which contained Y42A, S50A, S53A, S63A, T72A, T83A, T84A, and T95A mutations, was expressed and purified. *In vitro* MARK3 kinase assays were performed using varying amounts of ORF9b or ORF9b (p8M) proteins as in (**E**).

Data in (**F**) were presented as mean ± SEM in biological triplicates. *P* values in (**F**) were calculated by two-way ANOVA with Šidák's multiple comparisons tests. *** *P* < 0.001.

**Figure S11. MARK3 is a kinase responsible for the phosphorylation of ORF9b.**

(**A, B**) Structural models of ORF9b were predicted by AlphaFold 3. Overall cartoon representations of wild-type ORF9b (**A**, pink) and the phospho-deficient octuple mutant ORF9b (p8M; **B**, green) were shown. The eight phosphorylation sites in wild-type ORF9b (Y42, S50, S53, S63, T72, T83, T84, and T95) and their corresponding alanine substitutions in p8M were highlighted as sticks within a mesh representation. Neighboring residues within 5 Å of each indicated site are shown as sticks. The p8M model was superimposed onto wild-type ORF9b, and the Cα RMSD was calculated: the global and local RMSDs were 0.630 Å and 0.368 Å (residues within 5 Å of the mutated sites), respectively; the predicted ΔΔG and the maximum backbone-related energy deviation (max |Δbackbone|) were +10.828 REU and 0.820 REU, respectively, calculated by Rosetta cartesian_ddg.

(**C-J**) Eight single-site revertant mutants based on ORF9b (p8M) were *in vitro* expressed and purified. Size-exclusion chromatography (SEC) analyses of these recombinant proteins, including p8M-A42Y (**C**), p8M-A50S (**D**), p8M-A53S (**E**), p8M-A63S (**F**), p8M-A72T (**G**), p8M-A83T (**H**), p8M-A84T (**I**), and p8M-A95T (**J**), were conducted. All proteins were presented as both monomers and dimers.

(**K**) Coomassie blue staining of 2 μg of ORF9b (p8M)-based revertant mutants after SEC.

(**L**) *In vitro* MARK3 kinase assays using the ORF9b (p8M) mutant and the corresponding single-site revertant mutants as substrates.

(**M**) Schematic of *in vitro* PPM1A phosphatase activity assay. Phos-ORF9b proteins were produced via the MARK3 kinase assay, followed by concentration and buffer-exchange using ultrafiltration. Recombinant PPM1A and phos-ORF9b proteins were co-incubated in a phosphatase buffer. The released phosphates were detected with the malachite green phosphate detection reagent. Reactions were quantified by measuring absorbance at 630 nm.

(**N**) ORF9b proteins were phosphorylated via the *in vitro* MARK3 assay, followed by incubation with recombinant PPM1A (D239N) proteins to perform *in vitro* phosphatase experiment. Free phosphate was detected by Malachite Green Phosphate Detection Kit as in (**M**).

Data in (**L**) and (**N**) were presented as mean ± SEM in biological triplicates. *P* values in (**L**) were calculated by one-way ANOVA with Dunnett's multiple comparisons tests, while the *P* value in (**N**) was calculated by Student's *t*-tests. ns, not significant. *** *P* < 0.001.

**Figure S12. PPM1A catalytic activity is essential for the antagonism of antiviral gene expression.**

(**A**) ELISA quantification of IFN-β protein in the culture supernatants of HEK293T cells transfected with empty vector or with ORF9b from SARS-CoV-2 alone or in combination with PPM1A, followed by VSV infection, providing a protein-level validation of the RT-qPCR analysis of *IFNB1* shown in Figure 7G.

(**B-D**) RT-qPCR analysis of mRNA expression of *IFNB1* (**B**), *ISG15* (**C**), *ISG56* (**D**) in HEK293T cells overexpressed with ORF9b from SARS-CoV alone or in combination with PPM1A, followed by VSV infection.

(**E-G**) RT-qPCR analysis of mRNA expression of *IFNB1* (**E**), *ISG15* (**F**), *ISG56* (**G**) in HEK293T cells overexpressed with ORF9b from bat coronavirus alone or in combination with PPM1A, followed by VSV infection.

(**H-J**) HEK293T cells were co-overexpressed with ORF9b and either PPM1A (WT) or PPM1A (D239N) for 24 h, followed by VSV infection for 12 h. RT-qPCR analysis was performed to determine mRNA expression levels of *IFNB1* (**H**), *ISG15* (**I**), and *ISG56* (**J**).

(**K**) Uncropped full membrane images of the western blots corresponding to western blots presented in Figure 7J. Red boxes indicated the cropped regions shown in the respective figure panels. Molecular weight markers (kDa) were indicated in each membrane.

(**L**) ELISA quantification of IFN-β protein in the culture supernatants of A549-sgNT (non-targeting sgRNA control) and A549-sgPPM1A (PPM1A-knockout; sgPPM1A#1) cells, both transfected with SARS-CoV-2 ORF9b followed by VSV infection. This provided protein-level validation of the RT-qPCR analysis of *IFNB1* shown in Figure 7Q.

(**M-P**) Relative mRNA expression levels of *IFNB1* (**M**), *ISG15* (**N**), *ISG56* (**O**), and *IFITM1* (**P**) in A549 PPM1A knockout (PPM1A-KO) cells (sgPPM1A#1) overexpressed with ORF9b and either empty vector or PPM1A (WT), followed by VSV infection for 12 h.

Data in (**A-J**) and (**L-P**) were presented as mean ± SEM in biological triplicates. *P* values in (**A-G**) were calculated by two-way ANOVA with Šidák's multiple comparisons tests, while the *P* values in (**H-J**) and (**L-P**) were calculated by Student's *t*-tests. * *P* < 0.05, ** *P* < 0.01, *** *P* < 0.001.

**Figure S13. ORF9b potently antagonizes the innate immunity during early viral infection.**

(**A**) Purified SARS-CoV-2 virions were visualized by negative-staining transmission electron microscopy (TEM). Two representative viral particles were shown. Scale bar, 100 nm.

(**B-C**) Immunogold electron microscopy (iEM) analysis of SARS-CoV-2 virions using nucleocapsid (N)-specific (**B**) and ORF9b-specific (**C**) antibodies. Red arrows indicated antibody-labeled target proteins. Scale bar, 100 nm.

(**D**) Immunoblot detection of ORF9b in purified SARS-CoV-2 virions.

(**E**) Anti-GFP Co-IP analysis of GFP-tagged S, E, M, and N with HA-tagged ORF9b (SARS2).

(**F, G**) HEK293T cells were overexpressed with increasing amounts of MARK3, followed by infection with authentic SARS-CoV (**F**) or SARS-CoV-2 (**G**). Viral replication was quantified by measuring viral RNA copy numbers.

(**H**) HEK293T-sgNT and HEK293T-sgPPM1A cells were transfected with plasmids encoding human ACE2 for 24 h, followed by co-culture with indicated concentrations of SMIP-031 and SARS-CoV-2 JN.1 for 24 h. Viral RNA copies were quantified as in Figure 8L.

(**I**) Huh7 cells were co-cultured with indicated concentrations of PCC0208017 and SARS-CoV for 24 h. RT-qPCR was performed to determine the relative expression of SARS-CoV *N* mRNA.

(**J**) HEK293T-hACE2 cells were co-cultured with indicated concentrations of PCC0208017 and SARS-CoV-2 (JN.1) for 24 h. RT-qPCR analysis was performed to determine viral RNA copies within cells.

(**K**) Heatmap representation of the raw pathology scores assigned independently by three observers blinded to group allocation. Each row corresponds to one animal and each column to one observer; the score for each section is color-coded on a 0-4 scale (0, normal; 1, minimal; 2, mild; 3, moderate; 4, severe), as indicated. Animals are grouped by treatment (SMIP-031 0 mg/kg, SMIP-031 10 mg/kg, and SMIP-031 15 mg/kg).

(**L**) Line plot of the histopathological scores assigned by three independent, blinded observers for each animal (*n* = 5 mice per group; 15 animals in total). Inter-observer agreement was assessed using the intraclass correlation coefficient (ICC(2,3)), based on a two-way random-effects model with absolute agreement and the average of the three raters, which indicated excellent agreement among the three observers (ICC = 0.948, 95% CI 0.88-0.98). Each line represents one animal, and identical score combinations are superimposed.

Data in (**F-J**) were presented as mean ± SEM in biological triplicates. *P* values in (**F**), (**G**), (**I**), and (**J**) were calculated by one-way ANOVA with Dunnett's multiple comparisons tests, while *P* values in (**H**) were calculated by two-way ANOVA with Tukey's multiple comparisons tests. ns, not significant. * *P* < 0.05, ** *P* < 0.01, *** *P* < 0.001.

**Table S1. Complete catalog of 668 ORF9b homologous hits identified by tblastn.**

The table lists the hits with their sequence identifiers, alignment statistics, genomic properties, and source descriptions. Columns: qseqid, query sequence ID; sseqid, subject sequence ID; pident, percent of identical matches in the alignment; length, alignment length; mismatch, number of mismatches; gapopen, number of gap openings; qstart, start of alignment in query; qend, end of alignment in query; sstart, start of alignment in subject; send, end of alignment in subject; evalue, expect value; bitscore, bit score; genome_length_bp, subject genome length in base pairs; coverage (%), query coverage; description, subject sequence description.

The complete table is provided as a separate supplementary file.
